# Expanding chiral metamaterials for retrieving fingerprints via vibrational circular dichroism

**DOI:** 10.1038/s41377-023-01186-3

**Published:** 2023-06-25

**Authors:** Cheng Xu, Zhihao Ren, Hong Zhou, Jingkai Zhou, Chong Pei Ho, Nan Wang, Chengkuo Lee

**Affiliations:** 1 Department of Electrical and Computer Engineering, National University of Singapore, Singapore, 117583 Singapore; 2 Center for Intelligent Sensors and MEMS (CISM), National University of Singapore, Singapore, 117608 Singapore; 3 Institute of Microelectronics (IME), Agency for Science, Technology and Research (A*STAR), Singapore, 138634 Singapore; 4 NUS Graduate School for Integrative Science and Engineering Program (ISEP), National University of Singapore, Singapore, 117456 Singapore

**Keywords:** Mid-infrared photonics, Circular dichroism, Metamaterials, Infrared spectroscopy, Optical sensors

## Abstract

Circular dichroism (CD) spectroscopy has been widely demonstrated for detecting chiral molecules. However, the determination of chiral mixtures with various concentrations and enantiomeric ratios can be a challenging task. To solve this problem, we report an enhanced vibrational circular dichroism (VCD) sensing platform based on plasmonic chiral metamaterials, which presents a 6-magnitude signal enhancement with a selectivity of chiral molecules. Guided by coupled-mode theory, we leverage both in-plane and out-of-plane symmetry-breaking structures for chiral metamaterial design enabled by a two-step lithography process, which increases the near-field coupling strengths and varies the ratio between absorption and radiation loss, resulting in improved chiral light-matter interaction and enhanced molecular VCD signals. Besides, we demonstrate the thin-film sensing process of BSA and β-lactoglobulin proteins, which contain secondary structures α-helix and β-sheet and achieve a limit of detection down to zeptomole level. Furthermore, we also, for the first time, explore the potential of enhanced VCD spectroscopy by demonstrating a selective sensing process of chiral mixtures, where the mixing ratio can be successfully differentiated with our proposed chiral metamaterials. Our findings improve the sensing signal of molecules and expand the extractable information, paving the way toward label-free, compact, small-volume chiral molecule detection for stereochemical and clinical diagnosis applications.

## Abstract (web summary)

We demonstrate surface-enhanced vibrational circular dichroism spectroscopy for chiral mixture detection, enabled by MIR chiral metamaterial platform.

## Graphical abstract

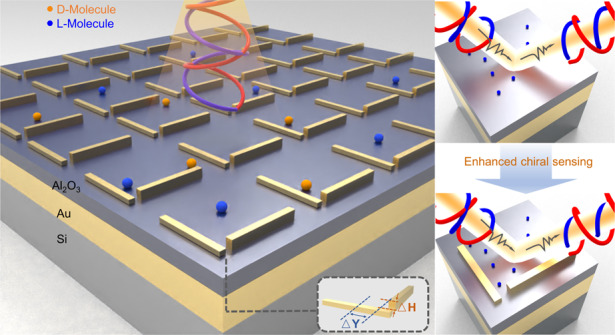

## Introduction

Enantiomers present different chemical properties, therefore, the detection of enantiomers plays an essential role in biomedical, pharmacological, and chemical analytical applications^1–3^. Due to the interaction between the chiral molecules and the spin momentum of the circularly polarized light, chiroptical spectroscopic solutions have been frequently reported for chirality sensing applications due to their instant response and accurate detection results^4,5^. The commonly used spectroscopy is circular dichroism (CD), which describes the absorption difference between left-handed circularly polarized light (LCP) and right-handed circularly polarized light (RCP)^6,7^. Considering the mismatch between molecule size and wavelengths, the strongest and most used CD signals are in ultraviolet, visible-light, and near-infrared regions. However, the molecular CD signals in these wavelengths cannot reflect the information of molecular chemical structures due to the lack of selectivity^8^, hindering the application for sensing different types of chiral molecules in mixtures. One intuitive method is to extend the wavelength ranges to mid-infrared (MIR) and leverage the vibrational information of molecules^9^, where the chemical bonds and functional groups reveal the identity of molecule structures, known as infrared fingerprints^10–12^. Such vibrational circular dichroism (VCD) spectroscopy leverages both the asymmetric response to circularly polarized light and IR fingerprints, enabling the identification of multidimensional structural information of molecules, especially protein secondary structures^13–15^. These secondary structures reveal the misfolding and aggregation of proteins and can lead to diseases like Alzheimer’s disease, Parkinson’s disease, and so on^16^. Compared with other methods of detecting such structures like atomic-force microscopy (AFM)^17^, nuclear magnetic resonance (NMR)^18^, and X-ray crystallography^19^, the VCD spectroscopic method provides a real-time and sensitive response of amide vibrational band (1700–1000 cm^−1^) for protein analysis, especially the amide I vibrational band (1700–1600 cm^−1^)^20^. However, the main limitation of VCD spectroscopy is the naturally weak signal, which is usually around 10^−5^ level, 3 magnitudes smaller than the UV-visible CD molecular signals^2^. Therefore, it is highly desired to develop enhanced VCD sensors for better calibration of the signals with smaller sample volumes, while exploring its potential for selective detection of chiral mixtures.

One of the best candidates to bridge the sensing limitation gap of VCD signals is MIR nanophotonic platform, which leverages ultra-confined optical field and resonant coupling^21^. MIR nanophotonics manipulate light in sub-wavelength regions and create surface-enhanced infrared absorption (SEIRA), enabling molecule sensing applications^9,22,23^, such as photonic crystals^24–27^, metamaterials^28–31^, waveguide sensors^32^, and nanomaterials^33^. Furthermore, combined with an unbalanced design framework, chiral nanostructures, also known as chiral metamaterials, can present anisotropic interaction with circularly polarized light^34^. Meanwhile, thanks to the interesting coupling phenomenon between enantiomers and chiral metamaterials, the enhanced optical chirality in the localized near-field region also enables stronger chiral light-matter interaction, raising research interests in valley-polarized photoluminescence^35^, drug screening^36^, and sensing applications^37,38^. However, only few attentions were paid to enhancing the weak VCD singals^21,39,40^. Besides, these works lack the design methodology and optimization process of the MIR chiral metamaterials. Although previous work has demonstrated the relationship between CD and non-radiative dissipation of MIR meta molecules using temporal coupled-mode theory (TCMT)^41^, the chiral light-matter interaction in the near field still lacks exploration.

In addition, regarding the fabrication process, chemical synthesis and self-assembly techniques have been widely proposed for manufacturing chiral nanostructures to achieve larger molecular CD signals^8^. Nevertheless, these methods can be time-consuming and less efficient for fabricating MIR chiral structures, as the dimensions of resonant structures increase to match longer wavelengths. Besides, lithography methods have also been utilized to pattern the chiral metamaterials, but also require a complex process to break the out-of-plane symmetry, which promises better performance^42^. Although previous work has proposed VCD sensing cavity that breaks the out-of-plane symmetry, the fabrication process can become a challenge^43^. Therefore, it is highly desired to develop MIR chiral metamaterials with well-explained theory and an easy-to-fabricate process to establish a feasible VCD sensing platform, which can expand the applications with retrieved molecule fingerprints.

In this work, we propose infrared chiral plasmonic metamaterials (IRCPMs) based on perpendicular positioned nanorods with a metal-insulator-metal (MIM) structure as a sensing platform. Like SEIRA, we propose Surface Enhanced Vibrational Circular Dichroism (SEVCD) spectroscopy for both enhanced molecular IR and CD signals sensing. As a design framework, we propose a loss engineering method supported by TCMT to design and optimize the chiral metamaterials by investigating loss ratio and near-field coupling coefficients, which influence the absorption difference between LCP and RCP electromagnetic waves. By tailoring these parameters, we optimize our chiral metamaterials by tuning both in-plane and out-of-plane asymmetric factors to achieve a larger VCD signal. Such proposed chiral metamaterials not only provide near-field enhancement through in-plane gaps, but also establish a 2.5D configuration to break the out-of-plane symmetry. In addition, our proposed IRCPMs only require a two-step e beam lithography and metal lift-off process. For the enhanced sensing process, our contribution is fourfold. First, we develop an enhanced VCD sensing platform consisting of chiral metamaterials that achieves an enhancement of 6 magnitudes compared with traditional VCD spectroscopy. Second, we illustrate the methodology of design and optimization of MIR chiral metamaterials leveraging temporal coupled-mode theory, which indicates the importance of near-field coupling coefficient and loss ratios, leading to the design of both in-plane and out-of-plane symmetry-breaking dimensions. Third, leveraging the mentioned sensing platform, we demonstrate the protein thin-film sensing process and achieve a lowest detection limit down to ~23 zeptomole level. Fourth, we for the first time report the enhanced VCD sensing process for mixed protein secondary structures with high selectivity from vibrational transitions. Compared with previous chiral mixture sensors^44,45^, our method also enables the selectivity of molecules from the vibrational transition in the absorption spectrum. Our results show a promising SEVCD chirality sensing platform for on-chip molecular identification from various species and low concentration for biomolecular and pharmacological analysis.

## Results

### Working mechanism of SEVCD sensing platform

**Fig. 1:**
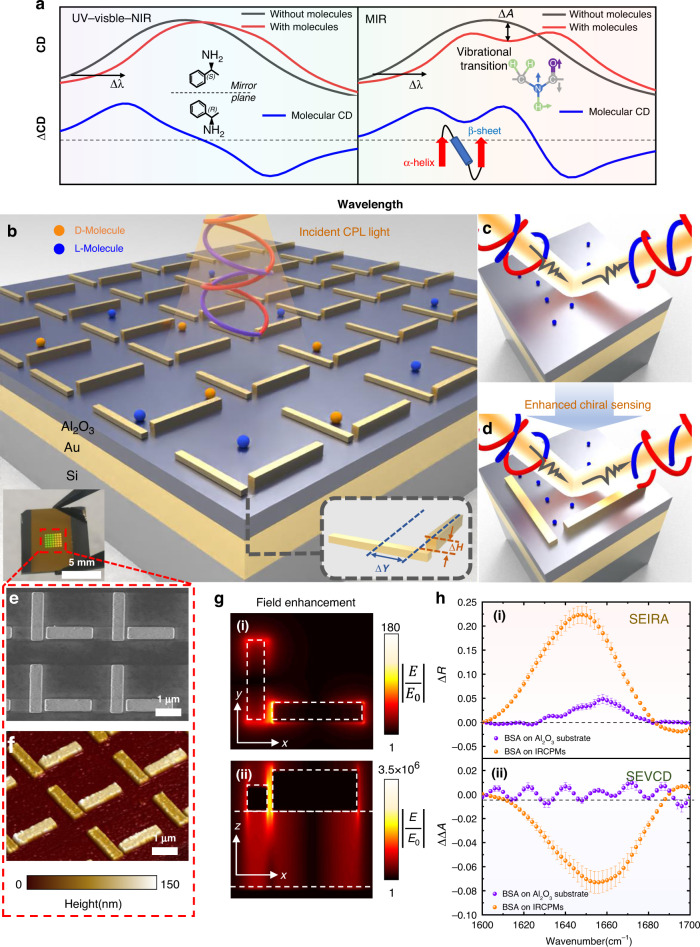
Operation principle of SEVCD spectroscopy using IRCPMs. **a** Illustration of enhanced CD sensing signal in UV, visible, NIR, and MIR regimes. For UV-visible-NIR wavelengths, the absorption peak of chiral molecules shows wavelength redshift. The molecular signal (blue curve) after removing the background signal indicates the molecular chirality, such as (S)-1-phenylethylamine and (R)-1-phenylethylamine. For MIR wavelengths, apart from the wavelength shift, MIR regime also provides vibrational transition of molecules. The relevant VCD signal could provide both vibration and chirality information, such as amide I vibration and secondary structures of proteins. **b** Schematic drawing of SEVCD platform. **c**, **d** Chiral molecule sensing improved by chiral structures, resulting in several magnitudes enhancement. **e** Scan electron microscope (SEM) image showing of IRCPMs. **f** Atomic-force microscopy (AFM) image showing of IRCPMs. The thicknesses of two presented nanorods are 85 nm and 108 nm. **g** Simulated field enhancement of IRCPMs on both x–y plane (i) and x–z plane (ii). The simulated wavenumber is 1650 cm^−1^. **h** Experimental BSA sensing results of enhanced VCD signal. For both SEIRA (i) and SEVCD (ii) spectroscopy. Compared with molecule signal on Al_2_O_3_ substrate, the signal is significantly improved when coated on IRCPMs

The concept of the SEVCD chiral molecule sensing platform is shown in Fig. 1. To explore the enhancing mechanism of VCD sensing, we propose reflective chiral metamaterials consisting of two orthogonal resonant modes. After analyzing the interaction between molecules and the near-field coupling between these resonant structures, we further optimize our structure for retrieving an enhanced far field molecule signal, and leverage such platform as biosensor for sensing protein secondary structures. We first illustrate the difference between traditional CD in UV-visible-NIR ranges and vibrational CD in MIR regime, as shown in Fig. 1a. Traditional absorptive CD spectrum detects the wavelength shift Δ*λ* when the sensors are coated with chiral molecules. After removing the background signal of the sensors, the molecular chirality can be recognized from the sign of the CD spectra. The signal level of typical enantiomers like (S)-1-phenylethylamine and (R)-1-phenylethylamine are around mdeg level. Unlike CD in short wavelength, mid-IR VCD spectroscopy not only illustrates molecular chirality, but also reveals the vibrational transition Δ*A*. Equipped with the multidimensional spectroscopic information, complex biomolecules structures can be effectively detected, such as amide I, amide II vibrations and secondary structures of proteins. However, the intrinsic VCD signal without enhancement is weak, which is only around μdeg level. Our IRCPM platform for enhancing the VCD signal is shown in Fig. 1b. The SEVCD metamaterial chip was made by integrating gold nanorods on top of the Al_2_O_3_-Au-Si substrate. The single Au nanoantenna was fabricated at the size of 1.7 μm × 0.4 μm through a two-step lithography process followed by deposition of different thicknesses of Au, and the Al_2_O_3_-Au layer is deposited on a dummy Si substrate, at the thickness of 200 nm and 100 nm, respectively (see “Materials and methods”: Sample fabrication and Supplementary Fig. S1). The chiral metamaterials coated with D- and L-chiral molecules present asymmetric signal absorption when LCP and RCP light are impinged onto the structures. As the bottom Au functions as a reflector, the incident light is either absorbed by the nanostructures or reflected to free space. Hence, by reading the reflection spectrum only, the absorption spectrum can be calculated as *A* = 1−*R*, where *R* and *A* represent the reflection and absorption coefficients. Leveraging our proposed chiral metamaterials, the reflective molecule signals are effectively enhanced by enlarging the absorption difference of the circularly polarized light, as shown in Fig. 1c, d. For better enhancing the chiral signals, we varied two important geometric factors here, labeled Δ*H* and Δ*Y*, which indicates the out-of-plane and in-plane asymmetries, respectively. The AFM and SEM image of the proposed IRCPM structures are shown in Fig. 1e, f, where different colors of these two nanorods indicate different deposition thicknesses of gold. The near-field simulation results illustrate that both in-plane and out-of-plane field enhancement is generated, where the strongest field confinement is observed in the nanogap region, which enables larger field intensity experienced by the molecules, as shown in Fig. 1g. To characterize the differences of the enhanced molecule signals with the removed signal of the chiral metamaterial background, we define two parameters, determined as:

1 $$\begin{array}{c}\Delta R={R}_{w.{molecule}}-{R}_{w/o.{molecule}}\end{array}$$

2 $$\begin{array}{c}\Delta \Delta A=\Delta {A}^{w.{molecule}}-\Delta {A}^{w/o.{molecule}}\end{array}$$

Where the subscript *w. molecule* and *w/o. molecule* represent *with molecule* and *without molecule*, respectively. The absorption difference Δ*A* is determined by:

3 $$\begin{array}{c}\Delta A={A}_{{LCP}}-{A}_{{RCP}}\end{array}$$

Leveraging these two parameters, we measured the enhanced molecule sensing signal for both IR and CD spectra, as shown in Fig. 1h. Coated with BSA proteins, the molecule signal on Al_2_O_3_ substrate is influenced by the noise from optical setup (see Supplementary Fig. S9), which is difficult to identify the BSA vibrational mode and its chirality, as shown in the purple curves in Fig. 1h(i) and Fig. 1h(ii). Fortunately, with our IRCPMs structures, both two molecule signals are amplified, which shows an amide I vibration around 1650 cm^−1^, and a negative chirality signal indicating the α-helical secondary structure. Such results indicate an effective and promising enhancement for both SEIRA and SEVCD spectroscopy.

### Design and optimization framework of chiral metamaterials

The resonant plasmonic structures could interact with light at certain wavelengths, forming a coupling system. In such a system, the radiative and absorption loss plays essential roles in the far-field spectrum. Our proposed IRCPM provides an approach to manipulating the absorption of circularly polarized light by tuning both the in-plane and out-of-plane asymmetric factors.

**Fig. 2:**
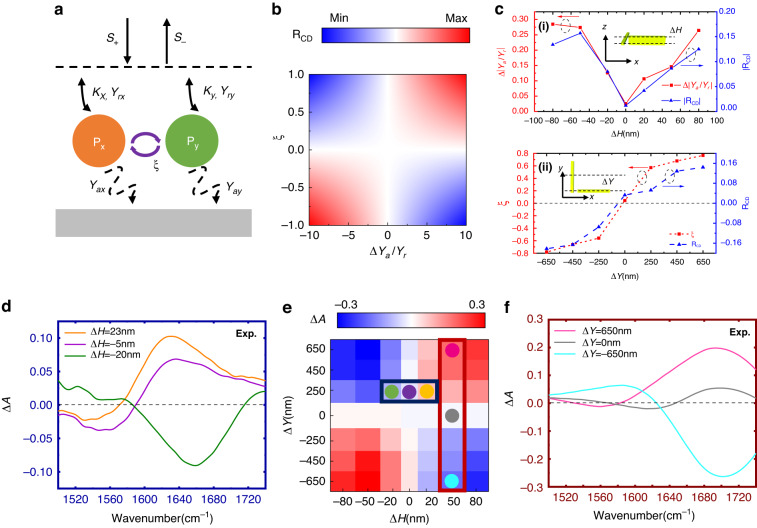
Design principle of proposed IRCPM. **a** Schematic drawing of the two-resonator TCMT model. **b** Calculation of the *R*_CD_ with respect to *ξ* and $\Delta \frac{{\gamma }_{a}}{{\gamma }_{r}}$. The color bar shows the normalized circular dichroism response. **c** Extracted *ξ* and $\Delta \frac{{\gamma }_{a}}{{\gamma }_{r}}$ of the designed metamaterials with out-of-plane (i) and in-plane displacements (ii). **d** Experimental results of the measured chiral metamaterials with varied thickness Δ*H*. **e** Simulated results of the circular dichroism with varied Δ*H* and Δ*Y*. The simulation wavenumber is set as 1667 cm^−1^. **f** Experimental results of the measured chiral metamaterials with varied positions on y-axis Δ*Y*

To illustrate our design principles, we use the TCMT to analyze the coupling system. The schematic of the TCMT model is shown in Fig. 2a. As our design is originated from double nanorod structures which are positioned perpendicularly, the model can be expressed as a two-resonator coupling system^46^:

4 $$\begin{array}{l}\displaystyle\frac{d}{{dt}}\left(\begin{array}{c}{P}_{x}\\ {P}_{y}\end{array}\right)=j\left(\begin{array}{cc}{\omega }_{x} & 0\\ 0 & {\omega }_{y}\end{array}\right)\left(\begin{array}{c}{P}_{x}\\ {P}_{y}\end{array}\right)\\\qquad\qquad\;\;-\left(\begin{array}{cc}{\gamma }_{{rx}}+{\gamma }_{{ax}} & j\xi \\ j\xi & {\gamma }_{{ry}}+{\gamma }_{{ay}}\end{array}\right)\left(\begin{array}{c}{P}_{x}\\ {P}_{y}\end{array}\right)+\left(\begin{array}{cc}{\kappa }_{x} & 0\\ 0 & {\kappa }_{y}\end{array}\right)\left(\begin{array}{c}{s}_{x}^{+}\\ {s}_{y}^{+}\end{array}\right)\end{array}$$

5 $$\begin{array}{c}\left(\begin{array}{c}{s}_{x}^{-}\\ {s}_{y}^{-}\end{array}\right)=\left(\begin{array}{cc}-1 & 0\\ 0 & -1\end{array}\right)\left(\begin{array}{c}{s}_{x}^{+}\\ {s}_{y}^{+}\end{array}\right)+\left(\begin{array}{cc}{\kappa }_{x} & 0\\ 0 & {\kappa }_{y}\end{array}\right)\left(\begin{array}{c}{P}_{x}\\ {P}_{y}\end{array}\right)\end{array}$$

6 $$\begin{array}{c}{\left(\begin{array}{c}{s}_{x}^{+}\\ {s}_{y}^{+}\end{array}\right)}^{{LCP}}=\displaystyle\frac{1}{\sqrt{2}}\left(\begin{array}{c}1\\ j\end{array}\right),{\left(\begin{array}{c}{s}_{x}^{+}\\ {s}_{y}^{+}\end{array}\right)}^{{RCP}}=\displaystyle\frac{1}{\sqrt{2}}\left(\begin{array}{c}1\\ -j\end{array}\right)\end{array}$$

where *P*_*x*_ and *P*_*y*_ are the mode amplitude of the plasmonic structure, *ω*_*x*_ and *ω*_*y*_ are the resonance frequency of the plasmonic structures oriented along the x and y axis, respectively. The radiative and absorptive losses are denoted as *γ*_*r*_ and *γ*_*a*_, while the near-field coupling coefficient between two resonances is written as *ξ*. *R* denotes the reflection spectrum, which is related to the amplitude of incident light (*S*^*+*^) and reflected light (*S*^*−*^). The far-field coupling coefficient is represented as *κ*. As the whole model is a two-resonator reflective coupling system, *κ* can be defined as $\sqrt{2{\gamma }_{r}}$. We could further obtain the reflective circular dichroism (see Supplementary Note 1 for details):

7 $$\begin{array}{c}{R}_{\rm{CD}}=\frac{4\xi (\frac{{\gamma }_{{ay}}}{{\gamma}_{{ry}}}-\frac{{\gamma }_{{ax}}}{{\gamma }_{{rx}}})({{\gamma }_{{rx}}{\gamma }_{{ry}}})^{\frac{3}{2}}}{{\left|{\xi}^{2}+\left[j\left({\omega }_{0}-{\omega }_{x}\right)+{\gamma }_{{rx}}+{\gamma }_{{ax}}\right]\left[j\left({\omega }_{0}-{\omega }_{y}\right)+{\gamma }_{{ry}}+{\gamma }_{{ay}}\right]\right|}^{2}}\end{array}$$

The *R*_CD_ represents the reflective CD in the far field, which has an opposite sign with Δ*A*. We use *R*_CD_ here to illustrate the CD signal induced only by the chiral metamaterials. From the numerator of the expression of *R*_CD_, two terms mainly determine the sign of CD, which are near-field coupling coefficient *ξ*, and the different ratio between the absorptive and radiative losses, denoted as $\Delta \frac{{\gamma }_{a}}{{\gamma }_{x}}=\frac{{\gamma }_{{ay}}}{{\gamma }_{{ry}}}-\frac{{\gamma }_{{ax}}}{{\gamma }_{{rx}}}$. We numerically calculated the relevant *R*_CD_ with varied *ξ* from −1 to 1 and varied $\Delta \frac{{\gamma }_{a}}{{\gamma }_{r}}$ from −10 to 10, as shown in Fig. 2b. As the nanoantennas are made of plasmonic nanorods and behave like dipoles, the absorption losses for the two modes are nearly equal. Therefore, two methods are proposed to enlarge the circular dichroism: create larger optical chirality in the near-field region to change *ξ* or construct the geometric asymmetry with different radiation losses for larger $\Delta \frac{{\gamma }_{a}}{{\gamma }_{r}}$. We create both out-of-plane and in-plane geometric variation, where Δ*H* is denoted as the thickness difference between the two nanoantennas and Δ*Y* represents the planar deviation on the y-axis. As Δ*H* and Δ*Y* are enlarged, the asymmetry on planar and vertical planes is increased, creating radiative losses and near-field coupling differences. This intuitive understanding is also confirmed by extracting *ξ* and $\Delta \frac{{\gamma }_{a}}{{\gamma }_{r}}$ in the TMCT model from the simulated reflection spectrum, as shown in Fig. 2c (see Supplementary Fig. S2). For both factors, the circular dichroism achieves near zero value when the *ξ* and $\Delta \frac{{\gamma }_{a}}{{\gamma }_{r}}$ is approaching zero. It should be noted that only the tuning of *ξ* could reach zero CD value, as the mean superchiral field can be zero by tuning the Δ*Y* into achiral structures. For $\Delta \frac{{\gamma }_{a}}{{\gamma }_{r}}$, the shape of these two nanoanatennas will be different by changing the thickness, while the planar shape is kept, which may decouple the near-field interaction with the loss changes^41^. In summary, a more intuitive understanding is that Δ*H* mainly controls the radiative loss of two plasmonic structures, while Δ*Y* mainly determines the near-field coupling between two orthogonal modes. This does not indicate the independent control for these two factors by varying the geometric parameters. However, leveraging this framework, the methodology for chiral sensor design can be well illustrated.

We make further numerical and experimental demonstrations with varied Δ*Y* and Δ*H*, as shown in Fig. 2d–f. In our simulated mapping of *R*_CD_, with varied Δ*Y* from −650 to 650 nm and varied Δ*H* from −80 to 80 nm, the *R*_CD_ presents similar hyperbolic-like distribution, which assembles the parameter mapping in Fig. 2b. This further illustrates the influence by the loss and near-field coupling. When Δ*Y* = 650 nm and Δ*H* = 80 nm, the circular dichroism reaches the largest value of 0.3. In addition, when Δ*Y* and Δ*H* are transferred from a positive value to a negative value, the CD is also turned into the opposite sign, illustrating the conversion of optical chirality. We experimentally validate these findings using fabricated devices with varied Δ*Y* from −650 to 650 nm and Δ*H* from −20 to 23 nm, as the orange, purple, and green curves shown in Fig. 2d, f. The results of orange and green curves are fitted well with the simulation results. However, for the purple curves where the Δ*Y* and Δ*H* are close to zero, the *R*_CD_ does not agree with the simulation well, which is due to the fabrication inaccuracy induced by the nonzeroΔ*Y* (see Supplementary Fig. S4) andΔ*H* (see Supplementary Fig. S11). Besides, it is also observable that when Δ*H* ~ 0, the *R*_CD_ is nonzero, as the planar structure with nonzero Δ*Y* is still chiral. However, the *R*_CD_ becomes near zero when Δ*Y* ~ 0, despite the value of Δ*H*. This further indicates that Δ*Y* controls the near-field coupling *ξ* while Δ*H* depends on the loss difference $\Delta \frac{{\gamma }_{a}}{{\gamma }_{r}}$, as the latter cannot reach zero value due to the asymmetry on x-axis and y-axis (see Supplementary Note 2 and Fig. S3 for details).

### Enhanced VCD measurement for chiral molecules

**Fig. 3:**
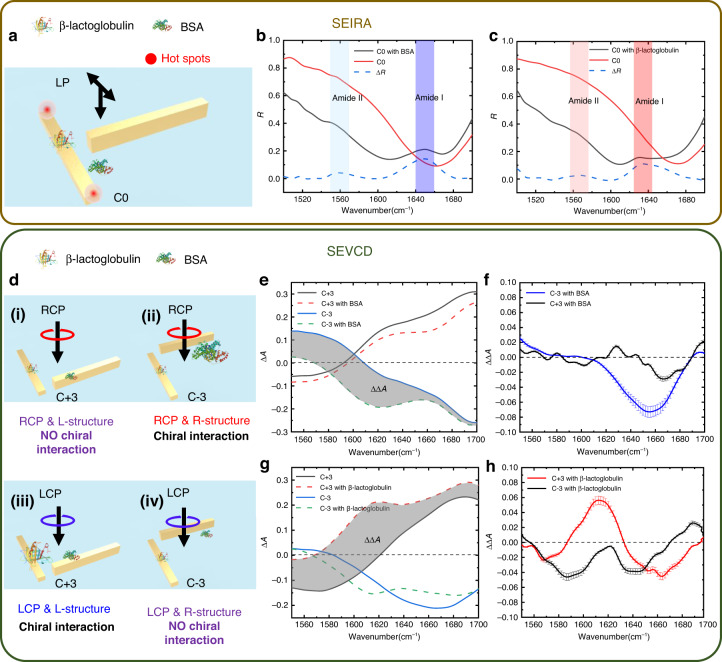
Experimental characterization of chiral proteins with chiral metamaterials. **a** Schematic drawing of the protein IR sensing. **b**, **c** Experimental results of BSA and β-lactoglobulin on C0 metamaterials. After removing the background of metamaterials, two enhanced peaks (blue curves) regarding amide I and amide II can be observed. **d** Schematic drawing of the protein CD sensing. **e**–**h** Experimental results of BSA and β-lactoglobulin on C−3 and C+3 metamaterials. Only when the chirality of protein matches the chirality of metamaterials, there will be interaction with light and CD peaks can be observed

For the sensing characterization, as the planar geometric asymmetry could provide more near-field coupling difference, we only use the metamaterials with fixed thickness difference at Δ*H* = −50 nm. Besides, we also label the structures with Δ*Y* difference from −650 to 650 nm as C+3, C+2, C+1, C0, C−1, C−2, and C−3 for better illustration (see Supplementary Information Fig. S4). To provide our unique sensing features of the proposed chiral metamaterials, we use the fabricated C+3, C−3, and C0 devices to demonstrate the signal acquisition process of both SEIRA and SEVCD spectroscopy, as shown in Fig. 3. We tested the sample of BSA and β-lactoglobulin prepared at a concentration of 250 ng/μL in DI water (see “Materials and methods”: Optical characterization). The protein solution of 2 μL was fetched and dropped onto the sample to form a thin film for optical characterization. As the vibrational wavelengths of BSA and β-lactoglobulin solution in DI water are around 6 μm^2^, we chose the chiral metamaterials with a length of 1.7 μm that have the same resonant wavelength of the protein (see Supplementary Note 3 and Fig. S5 for details). The results for SEIRA spectroscopy are shown in Fig. 3a–c. The C0 metamaterial presents a resonant frequency near the wavenumber 1640 cm^−1^, generating hotspots at each edge of the nanoantenna. When BSA was coated onto the surface, two reflection peaks arose at the wavenumber 1560 and 1650 cm^−1^, indicating the vibrational mode for amide II and amide I of BSA, respectively, as the black and red curves shown in Fig. 3b. To remove the background signal of metamaterials, we subtract these two signals, the results is shown as the blue curve in Fig. 3b. The IR absorption peak of amide II is weaker than amide I, which is because of the wavelength mismatch between the metamaterials and the amide II fingerprint (see Supplementary Fig. S16). Similarly, we implement the process for the β-lactoglobulin signal, as shown in Fig. 3c, where two peaks are at wavenumber 1565 and 1633 cm^−1^. We have also demonstrated stronger amide II resonance using longer nanorods (see Supplementary Fig. S15). Compared to the IR signals of these two proteins, the different vibrational modes of the amide I illustrate the identity of each molecule. For the SEVCD spectrum, the schematics showing the sensing principles are shown in Fig. 3d. Chiral structures C+3 and C−3 are used to detect the optical chirality difference as they could provide larger circular dichroism. As the absorption signal for amide II is much smaller than amide I for both proteins, while our 1/4 waveplate only covers a narrow band (λ_0_ = 6 μm, Δλ = 400 nm), we only chose the peaks for amide I for SEVCD demonstration. Coated with proteins, the C+3 structure absorbs more LCP light and has no interaction with RCP light. Hence, for β-lactoglobulin, the CD signal can be enhanced by the C+3 structure. Similarly, C−3 can enhance the CD signal for BSA. The Δ*A* results are shown in Fig. 3e, g. It can be noticed that with the proteins, the initial CD signal showed both wavelength shift and absorption peaks, this is because of the index change by the protein thin film as well as the IR absorption signals. We also subtract the signal to remove the influence of the metamaterials. The ΔΔ*A* results are shown in Fig. 3f, h. The molecule CD signal is effectively enhanced when the chirality matches with the structural chirality. Thereby, we could observe a magnified BSA signal with C−3 metamaterials and β-lactoglobulin signal with C+3 metamaterials. Moreover, the absorption peaks agree with the IR fingerprints of these two molecules, indicating the featured information for both chiral molecules. Thereby, we successfully demonstrate the enhanced molecule signals via both SEIRA and SEVCD spectroscopy.

**Fig. 4:**
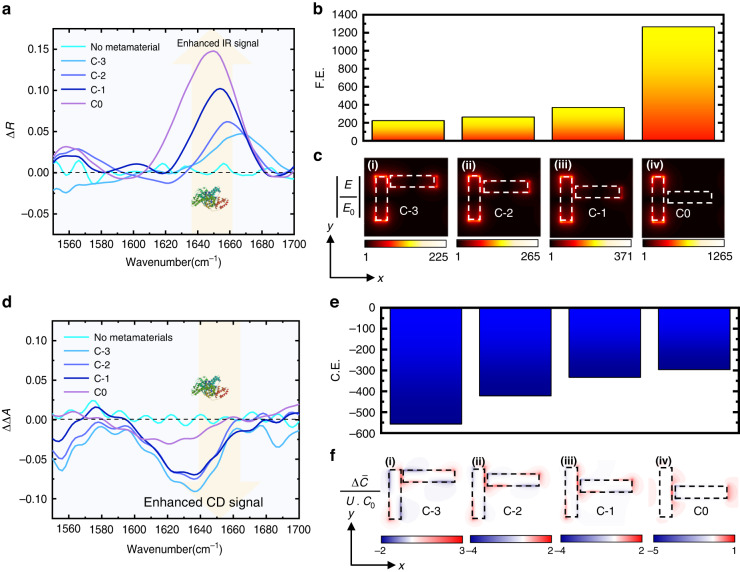
Near-field illustration of the CD and IR enhancement. **a** Experimental results of the Δ*R* signal on different chiral metamaterials. **b**, **c** Simulated near-field enhancement from C−3 to C0 metamaterials. **d** Experimental results of the ΔΔ*A* signal on different chiral metamaterials. **e**, **f** Simulated average chiral enhancement from C−3 to C0 metamaterials

We also experimentally analyzed the enhancement performance for different chiral metamaterials and compared them with the near-field simulation results to illustrate the enhancing mechanisms, as shown in Fig. 4. All the chiral metamaterials used in this demonstration have the same gap of 100 nm, as 100 nm gaps could provide larger sensing performance than 200 nm gaps. For smaller gaps, the performance is limited by the fabrication limit and strong coupling induced Rabi-splitting (see Supplementary Fig. S8 and Supplementary Note 3). We coated BSA onto the chiral metamaterials with a concentration of 250 ng/μL. Four different chiral metamaterials are measured to obtain both the IR absorption and CD signals, labeled C−3, C−2, C−1, and C0, as shown in Fig. 4a, d. The Δ*R* increases when the device is varied from C−3 to C0, where C0 presented the largest signal of around 0.15. This enhanced molecule signal is 3 times larger compared with C−3 metamaterials, and around 15 times larger than the pure reflective molecule signal without being improved by any metamaterials. We use FDTD simulation to simulate the electrical near-field distribution of these four metamaterials, as shown in Fig. 4b, c. As the polarization is linear along the left nanorod, the dipole moment along the y axis is generated. The field enhancement *F.E*. is determined by:

8 $$\begin{array}{c}F.E.=\max \left(\left|\frac{E}{{E}_{0}}\right|\right)\end{array}$$

Where *E*_0_ is the near-field intensity without metamaterials. The *F.E*. of C0 achieved around 1265, which is 5 times larger than the C−3 metamaterial, suggesting that molecules located at such metamaterial could experience larger near-field enhancement compared with other structures. Besides, from the reflective spectrum, the C0 metamaterial has the highest Q factor for the resonant peak (around 11.9), indicating larger field confinement (see Supplementary Fig. S7 and Supplementary Table S2). Therefore, we conclude that the C0 structures with less asymmetry and higher field enhancement could provide the highest IR absorption signal.

For the chirality enhancement, different from the IR sensing results, the C−3 has the largest CD signal than the others, which agreed with the optimized results shown in Fig. 2. We also simulate the optical chirality of the four structures. As the nanoantennas are of varied thicknesses, we implement multiple monitors from the bottom surface to the top surface of the antenna to obtain the average optical chirality for the whole structure. The differential optical chirality is determined by:

9 $$\begin{array}{c}{C}_{i}=-\frac{{\varepsilon }_{0}\omega }{2}\left({{{\boldsymbol{E}}}_{{\boldsymbol{i}}}}^{* }\cdot {{\boldsymbol{B}}}_{{\boldsymbol{i}}}\right)\end{array}$$

10 $$\begin{array}{c}\bar{C}=\mathop{\sum }\limits_{i=1}^{N}\frac{{C}_{i}}{N}\end{array}$$

11 $$\begin{array}{c}\Delta \bar{C}={\bar{C}}_{{LCP}}-{\bar{C}}_{{RCP}}\end{array}$$

where *ε*_0_ is the permittivity in vacuum, *ω* is the angular frequency of the circular polarized light, ***E*** and ***B*** are the intensities of electric and magnetic fields, respectively. The subscript *i* is the number of the electric field monitor, and *N* is the total number of the monitors along the vertical direction, which can be arbitrarily chosen depending on the thickness difference Δ*H*. The reason for placing multiple monitors is to calculate the total superchiral field distribution of the thickness-varied nanorod structure. As the molecules are randomly localized around the chiral metamaterials, we further calculate the average value of the optical chirality as a mean superchiral field that a molecule could experience. To eliminate the influence of the field enhancement, we further divide the total power of the plasmonic resonator, denoted as *U*. The determination of chiral enhancement *C.E*. provided by each structure is determined by:

12 $$\begin{array}{c}C.E.=\iint \frac{\triangle \bar{C}}{U\cdot {C}_{0}}{dxdy}\end{array}$$

where *C*_0_ represents the optical chirality without the chiral metamaterials in free space. The integral denotes the total superchiral field on one unit cell, which means the chiral enhancement that molecules could experience for each unit structure. The near-field images are shown in Fig. 4e, f. The localized field of the C−3 structure showed the highest enhancement and asymmetry, followed by C−2 and C−1. For the C0 structure, although the field intensity is larger, the mean chirality is small and thus, difficult to interact with chiral molecules (see Supplementary Note 4 and Fig. S6 for details). Hence, for right-handed chiral molecule sensing, C−3 structure with larger asymmetry could provide higher enhancement of superchiral field, enabling larger molecule signal compared with other structures. Although we only present the results of right-handed structures as a demonstration of enhanced BSA sensing, such analysis and results are also applicable for left-handed structures, where the C+3 structure has larger enhancement for enhanced β-lactoglobulin sensing.

### Chirality determination of chiral molecules with different concentration

**Fig. 5:**
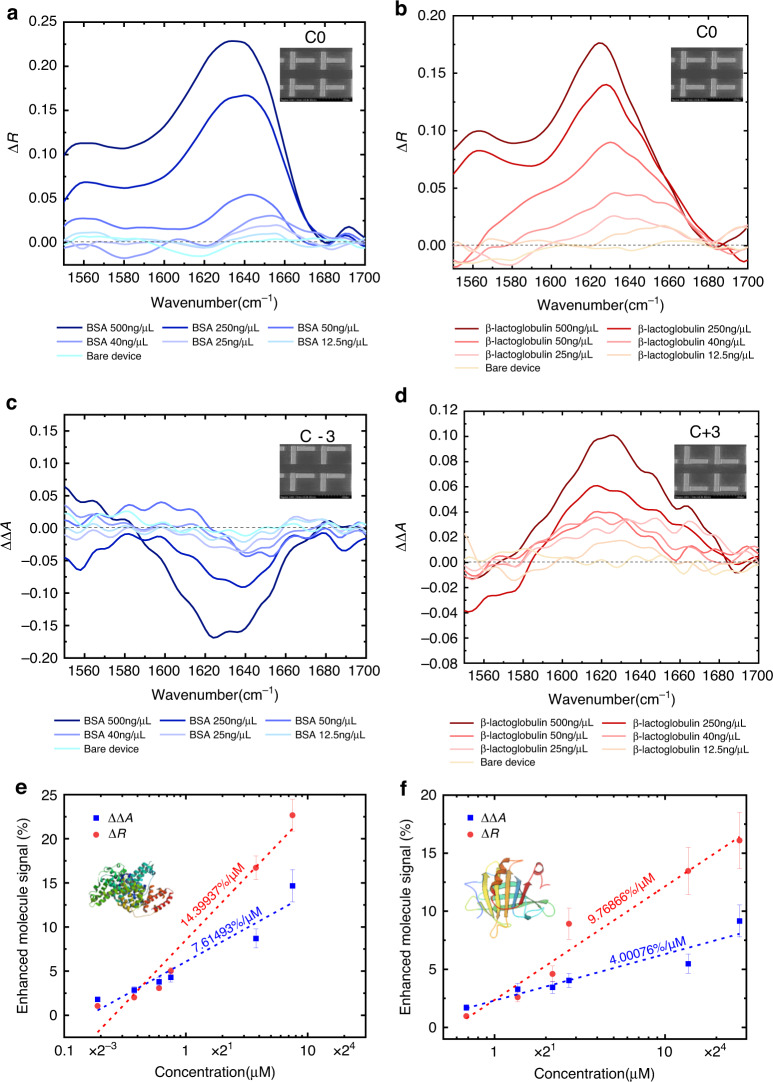
Experimental characterization of proteins with varying concentrations. **a**, **b** Experimental results of enhanced IR signal from BSA and β-lactoglobulin on C0 metamaterials, with scanning electron microscopy image of the C0 device. **c**, **d** Experimental results of enhanced CD signal from BSA and β-lactoglobulin on C−3 and C+3 metamaterials with scanning electron microscopy image of the C−3 and C+3 devices, respectively. **e**, **f** Experimental enhanced molecule signals versus the protein concentrations. Dashed lines show the fitting curves

We have further varied the concentration of BSA and β-lactoglobulin solutions to demonstrate the limit of detection of our device. Such capability is crucial for developing quantitively monitoring chemical synthesis processes. As illustrated in Fig. 4, we use the C0 structure for SEIRA detection of both proteins, while C+3 and C−3 are used for the SEVCD spectrum of β-lactoglobulin and BSA, respectively. The detailed measurement results can be found in Fig. 5. We first prepared both BSA and β-lactoglobulin solutions with 1000 ng/μL concentration and dilute it with DI water to obtain 500, 250, 50, 40, 25, and 12.5 ng/μL concentrations. We use the same sample for the measurement of each concentration, with cleaning steps between each test to avoid signal interference (see “Materials and methods”: Optical characterization). We first use the C0 structure for the detection of IR absorptive signals for varying concentrations, as shown in Fig. 5a, b. For both proteins, the absorptive signal grew with increasing concentration at the molecule vibrational wavelengths. Even at the smallest concentration of 12.5 ng/μL, a signal contrast of around 0.015 can be observed with a small volume of 1 μL (see Supplementary Fig. S9). Compared with traditional VCD signal for BSA and β-lactoglobulin, which required a concentration of 20 mg/100 μL to obtain a spectroscopy signal of 10^−5^ level. Our platform effectively enhances the molecule signal by 6 magnitudes^2^. Moreover, this has not reached the detection limit of our device due to the intrinsic noise of the integrated laser setup. The results for SEVCD are shown in Fig. 5c, d. The chiral response of BSA and β-lactoglobulin of opposite signs also showed an opposite chirality. We further plot the IR and CD signals with concentrations in Fig. 5e, f. It can be observed that for both proteins, the increasing rate of SEIRA (14.39937%/μM and 9.76866%/μM) is larger than SEVCD (7.61493%/μM and 4.00076%/μM), indicating the advantage of using IR signals for quantified detection, where a maximum sensitivity of 14.39937%/μM is achieved for the SEIRA detection of BSA.

### Detection of chiral mixture

**Fig. 6:**
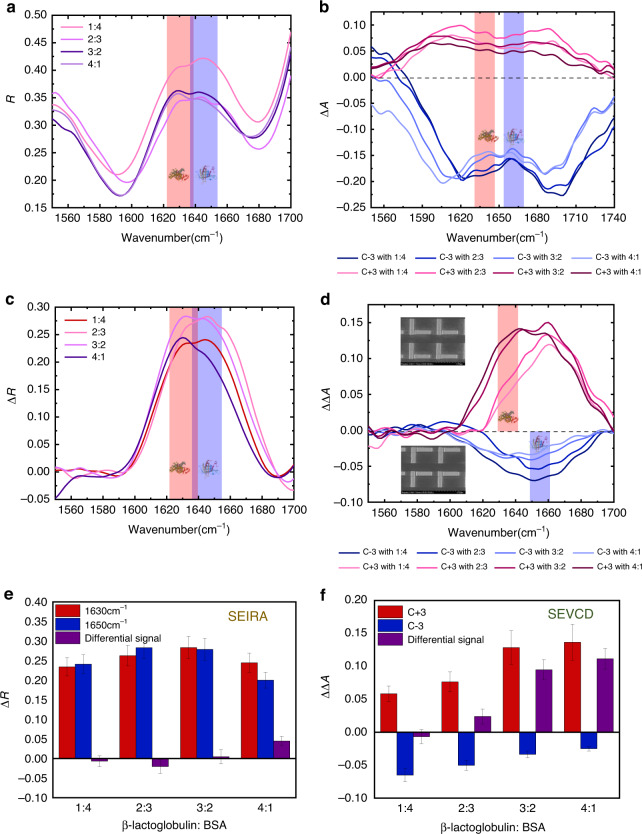
Experimental demonstration for chiral mixture sensing. **a**, **b** Reflection and circular dichroism spectra for mixed BSA and β-lactoglobulin. **c**, **d** Enhanced IR and CD signal for mixed BSA and β-lactoglobulin sensing with 4 different concentration ratios. For IR sensing, C0 metamaterial is used. For CD sensing, C−3 and C+3 metamaterials are used. **e** Extracted maximum signal difference from the IR spectrum at two resonant wavelengths. **f** Extracted maximum signal difference from the CD spectrum

We finally experimentally demonstrated the detection of mixed chiral compounds using our proposed metamaterials. The feasibility of distinguishing different chiral structures in mixtures could enlarge the applications for our chiral sensors, such as in the pharmaceutical industry, where chiral impurities lead to side effect or opposite effect in drug production^3^. We mixed 10 μL of each BSA and β-lactoglobulin solutions of 250 ng/μL concentration into four different ratios, labeled 1:4, 2:3, 3:2, and 4:1, respectively. We also dropped 2 μL of each solution onto the chiral metamaterials to detect the signal difference of each SEIRA and SEVCD spectra, as shown in Fig. 6. For the IR spectrum, we could visualize two closed reflection peaks at wavenumber 1630 and 1650 cm^−1^, indicating the fingerprint of β-lactoglobulin and BSA molecules, respectively (Fig. 6a). We further plot the Δ*R* signals for each protein and calculate the signal difference in Fig. 6c, e. At this step, the identities of these two proteins are revealed according to the vibrational wavelength. However, the contrast of these two molecules regarding the mixed ratio is not high enough. For example, the signal even showed a decreased change when the ratio changed from 1:4 to 2:3. The reason for the abnormal signal may be because of the overlap between two wavenumbers. As our proposed IRCPM required a proper ratio between the absorption loss and the radiative, and the selectivity is determined by the absorption loss of the plasmonic structures, the sensitivity of closed wavelength ranges may be affected by the overlapped signals. We further apply the CD spectrum for the mixed proteins’ compounds, which expands its capability of differentiating the closed signals in the spectrum, as shown in Fig. 6b, d, f. Leveraging the chiral structures mentioned above, we distinguish the positive and negative chiral signals and compare the intensities for each mixed ratio. As the molecule mass of β-lactoglobulin is larger than BSA, the signal of positive chiral structure is slightly larger than negative signals. From the shown differential data, the chirality showed an increased tendency from negative to positive as the BSA ratio becomes larger. This demonstration indicates that for the mixed chiral structure sensing, our platform could provide the SEIRA spectrum to identify the molecule without labels by specifying the IR absorption peaks, followed by the SEVCD spectrum to quantitatively determine the CD signals. Combining the two spectra, the chiral metamaterials could enable the detection of unlabeled chiral mixtures with similar absorption peaks at a closed ratio. Such demonstration indicates the potential for label-free detection of mixed molecules with similar IR fingerprints and different chirality. We believe that our proposed mid-IR chiral sensing platform paves the way to the state-of-the-art applications in drug delivery^47,48^, biomedical detection^8^, healthcare^45^, and clinical diagnosis^49^.

## Discussion

In conclusion, we have experimentally demonstrated a platform for SEVCD chiral molecule detection via reflective chiral metamaterials. Compared with traditional SEIRA spectroscopy, SEVCD not only reveals the molecule identity through IR fingerprints, but also provides structural information of chiral molecules. In chiral metamaterial design, we leverage the loss engineering process to optimize the IRCPM nanostructures by enlarging both in-plane and out-of-plane asymmetry (Δ*H* and Δ*Y*), which influences the near-field coupling and loss($\Delta \frac{{\gamma }_{a}}{{\gamma }_{r}}$ and *ξ*), respectively. Such results illustrate the design methodology for chiral metamaterial sensor design, which have been theoretically and experimentally validated using two-step lithographically fabricated devices. Furthermore, we use BSA and β-lactoglobulin, two proteins with similar vibrational modes and different optical chirality at the mid-IR wavelengths, and experimentally measured enhanced Δ*R* and ΔΔ*A* molecule signals for C0 and C−3 structures, respectively. This enhancement is also theoretically explained by the near-field enhancement. In the next step, we varied the concentration of each protein to measure the sensitivity and obtained a highest sensitivity of 14.4%/μM for SEIRA detection of BSA. In addition to that, we have achieved the chiral signal with a low concentration of 12.5 ng/μL and a small sample volume of 1 μL, which show a 6-magnitude improvement compared with traditional VCD sensing performance. Such volume is sufficient to detect the chirality of proteins with ~23 zeptomoles of BSA in our sensing area, which corresponds to ~14,000 molecules per metamaterial array of 200 × 200 μm^2^ footprint (see “Materials and methods”: Estimation of detection limit). This value is limited by the noise of optical characterization equipment, which is induced by the coherence of the QCL laser, and has not reached the physical limitation of our device (see Supplementary Note 5 and Fig. S9). Apart from protein secondary structure sensing, we have also demonstrated enantioselective sensing using L-glucose and D-glucose using the same platform (see in Supplementary Fig. S17 and Supplementary Note 6). The results show a promising enhanced VCD signal for the chiral isomers, indicating the potential application of our platform in stereochemistry areas. Moreover, we have further experimentally demonstrated, for the first time, the distinguishment of the two proteins in a ratio-dependent mixture using enhanced VCD spectroscopy (see Supplementary Fig. S18 and Supplementary Table S1). This detection process not only leverages molecule fingerprint from the IR spectrum and molecule chirality from the CD spectrum, but also solves the signal overlap and the label-attached problems for both spectra. Benefiting from the high detectivity, low sample consumption, and feasible large-scale fabrication process, our IRCPM enables a promising chiral sensing platform for ultrasmall-volume sensing and label-free chiral mixture detection, which opens up a new avenue for chemical or biomedical applications such as the study and analysis of chiral nanostructures in dynamic reactions.

## Materials and methods

### Sample fabrication

Dummy silicon wafer was sonicated in acetone for 3 min and then rinsed in IPA followed by nitrogen drying. After drying, 5 nm thick Ti, 100 nm thick Au, and 200 nm Al_2_O_3_ was deposited on to the Si wafer in sequence by e-beam evaporator (AJA Ebeam evaporator). Next, positive e-beam resist PMMA was coated onto the substrate with a speed of 4000 rpm for 1 min. Since the Al_2_O_3_ substrate was insulating, a thin conducting polymer file ESpacer was also spin-coated with a speed of 4000 rpm for 40 s. After being exposed by E-beam lithography (JEOL JBX-6300FS) to pattern the first nanoantenna, the sample was first immersed into DI water for 15 s to remove the ESpacer, then developed in a mixture of MIBK/IPA (1:3) for 40 s, and finally rinsed in IPA for 30 s. After that, 5 nm thick Cr and 60 nm Au were deposited on to the sample using thermal evaporator (Lesker NANO 36), followed by a lift-off process in acetone at 65 °C for 45 min. After that, another e-beam lithography and thermal evaporation for 5 nm Cr and 120 nm Au was processed to fabricate the second nanoantenna, followed by another lift-off process. The different thickness of the nanorods is characterized by AFM (Bruker Dimension FastScan) (see Supplementary Fig. S11).

### Estimation of detection limit

To quantify how much protein molecules are covering our chiral metamaterial array, we measured the surface microscopy image using AFM (Bruker Dimension FastScan). We select one of our samples (Δ*H* = 20 nm) and first measured the surface without nanoantennas. After that, we dropped 2 μL of 250 ng/μL BSA solution onto the sample to cover all the metamaterial arrays. After the solution was dried, we took another AFM image and compared it with the previous image (see Supplementary Fig. S12). Using image-processing software (NanoScope Analysis), we estimate that approximately 35 ± 9 molecules cover an area of 10 × 10 μm^2^. The whole sensing area is 200 × 200 μm^2^ on the sample, corresponding to ~14,000 molecules or ~23.26 zeptomoles. The dimension of BSA is estimated based on 14 nm × 4.1 nm × 4.1 nm determined by birefringence relaxation studies^50^.

### Optical characterization

The chiral metamaterial was characterized by Spero^®^ Chemical Imaging Microscope (see Supplementary Fig. S10). One 1/4 waveplate (Edmund Optics, #85-120) was used to change the linearly polarized light to circularly polarized light. The IR image of IRCPM array as well as the optical microscope image of C−3 and C+3 devices can be found in Supplementary Fig. S13 and Supplementary Movie 1. For the protein sensing measurement, β-lactoglobulin and Bovine Serum Albumin were used (Sigma-Aldrich, product L3908 and A7030). The proteins were dissolved in DI water and formed a solution with different concentrations, ranging from 12.5 to 1000 ng/μl. The chiral metamaterials were used for multiple measurements. For each measurement, protein solution was fetched using a micropipette and dropped onto the metamaterials. The measurement was conducted after 15 min when the droplet was dried. After each measurement, the sample was immersed into DI water for 30 min and then cleaned by nitrogen drying. The reproducibility of our device is discussed as shown in Supplementary Fig. S14. For each repeated test, we use the protein solution with same concentration and same sample volume before coated on the device substrate.

### Numerical simulations

The simulation was performed using a 3D FDTD method (Lumerical-FDTD). In the simulation, the complex refractive index of Au, Al_2_O_3_, and Si from Palik et al. is used. The thickness of Si is set as infinite. The simulation was performed on a unit cell with periodic conditions.

## Supplementary information

Supplementary Information

Supplementary Movie 1
