# Supplementary material for: Expanding chiral metamaterials for retrieving fingerprints via vibrational circular dichroism
Source: Light Sci Appl. 2023 Jun 25;12:154. doi: 10.1038/s41377-023-01186-3 (PMC10290984; doi:10.1038/s41377-023-01186-3)
Supplement: Supplementary file 1 — Supplementary Information [file 41377_2023_1186_MOESM1_ESM.docx]

Supplementary Information for

**Expanding chiral metamaterials for retrieving fingerprints via vibrational circular dichroism**

Cheng Xu, Zhihao Ren, Hong Zhou, Jingkai Zhou, Chong Pei Ho, Nan Wang and Chengkuo Lee^*^

*Corresponding author: elelc@nus.edu.sg

**Supplementary Note 1 to 6**

**Supplementary Figs. S1 to S18**

**Supplementary Tables S1 to S2**

**Supplementary Note 1: Derivation of TCMT model**

The model is expressed as:

$\begin{aligned} \frac{d}{dt}\left( \begin{matrix} P_{x} \\ P_{y} \end{matrix} \right)=j\left( \begin{matrix} \omega_{x} & 0 \\ 0 & \omega_{y} \end{matrix} \right)\left( \begin{matrix} P_{x} \\ P_{y} \end{matrix} \right)-\left( \begin{matrix} \gamma_{rx}+\gamma_{ax} & j\xi\\ j\xi& \gamma_{ry}+\gamma_{ay} \end{matrix} \right)\left( \begin{matrix} P_{x} \\ P_{y} \end{matrix} \right)+\left( \begin{matrix} \kappa_{x} & 0 \\ 0 & \kappa_{y} \end{matrix} \right)\left( \begin{matrix} s_{x}^{+} \\ s_{y}^{+} \end{matrix} \right)\#\left( S1 \right) \end{aligned}$

The mode amplitude term $\left( \begin{matrix} P_{x} \\ P_{y} \end{matrix} \right)$ can be expressed as:

$\begin{aligned} \left( \begin{matrix} P_{x} \\ P_{y} \end{matrix} \right)=\frac{\left( \begin{matrix} j\left( \omega_{0}-\omega_{y} \right)+\gamma_{ry}+\gamma_{ay} & -j\xi\\ -j\xi& j\left( \omega_{0}-\omega_{x} \right)+\gamma_{rx}+\gamma_{ax} \end{matrix} \right)\left( \begin{matrix} \kappa_{x} & 0 \\ 0 & \kappa_{y} \end{matrix} \right)\left( \begin{matrix} s_{x}^{+} \\ s_{y}^{+} \end{matrix} \right)}{\xi^{2}+\left[ j\left( \omega_{0}-\omega_{x} \right)+\gamma_{rx}+\gamma_{ax} \right]\left[ j\left( \omega_{0}-\omega_{y} \right)+\gamma_{ry}+\gamma_{ay} \right]}\#\left( S2 \right) \end{aligned}$

Substituting the $\left( \begin{matrix} P_{x} \\ P_{y} \end{matrix} \right)$, we could obtain the relationship between the input and output parameters:

$\begin{aligned} \left( \begin{matrix} s_{x}^{-} \\ s_{y}^{-} \end{matrix} \right)=\left( \begin{matrix} -1 & 0 \\ 0 & -1 \end{matrix} \right)\left( \begin{matrix} s_{x}^{+} \\ s_{y}^{+} \end{matrix} \right)+\frac{\left( \begin{matrix} 2\gamma_{rx}\left[ j\left( \omega_{0}-\omega_{y} \right)+\gamma_{ry}+\gamma_{ay} \right] & -2j\xi\sqrt{\gamma_{rx}\gamma_{ry}} \\ -2j\xi\sqrt{\gamma_{rx}\gamma_{ry}} & 2\gamma_{ry}\left[ j\left( \omega_{0}-\omega_{x} \right)+\gamma_{rx}+\gamma_{ax} \right] \end{matrix} \right)\left( \begin{matrix} s_{x}^{+} \\ s_{y}^{+} \end{matrix} \right)}{\xi^{2}+\left[ j\left( \omega_{0}-\omega_{x} \right)+\gamma_{rx}+\gamma_{ax} \right]\left[ j\left( \omega_{0}-\omega_{y} \right)+\gamma_{ry}+\gamma_{ay} \right]}\#\left( S3 \right) \end{aligned}$

As our devices are composed of MIM structures, where a metal reflector is deposited at the bottom, the reflectivity matrix can be determined as:

$\begin{aligned} \bar{r}=\left( \begin{matrix} s_{x}^{-} \\ s_{y}^{-} \end{matrix} \right)=\frac{\left( \begin{matrix} -\xi^{2}-\left[ j\left( \omega_{0}-\omega_{y} \right)+\gamma_{ry}+\gamma_{ay} \right]\left[ j\left( \omega_{0}-\omega_{x} \right)-\gamma_{rx}+\gamma_{ax} \right] & -2j\xi\sqrt{\gamma_{rx}\gamma_{ry}} \\ -2j\xi\sqrt{\gamma_{rx}\gamma_{ry}} & -\xi^{2}-\left[ j\left( \omega_{0}-\omega_{x} \right)+\gamma_{rx}+\gamma_{ax} \right]\left[ j\left( \omega_{0}-\omega_{y} \right)-\gamma_{ry}+\gamma_{ay} \right] \end{matrix} \right)\left( \begin{matrix} s_{x}^{+} \\ s_{y}^{+} \end{matrix} \right)}{\xi^{2}+\left[ j\left( \omega_{0}-\omega_{x} \right)+\gamma_{rx}+\gamma_{ax} \right]\left[ j\left( \omega_{0}-\omega_{y} \right)+\gamma_{ry}+\gamma_{ay} \right]}\#\left( S4 \right) \end{aligned}$

The circular dichroism is defined by the subtraction between LCP and RCP light, where the electric field of LCP and RCP light is given by:

$\begin{aligned} \left( \begin{matrix} s_{x}^{+} \\ s_{y}^{+} \end{matrix} \right)^{LCP}=\frac{1}{\sqrt{2}}\left( \begin{matrix} 1 \\ j \end{matrix} \right),\left( \begin{matrix} s_{x}^{+} \\ s_{y}^{+} \end{matrix} \right)^{RCP}=\frac{1}{\sqrt{2}}\left( \begin{matrix} 1 \\ -j \end{matrix} \right)\#\left( S5 \right) \end{aligned}$

And the *R_CD_* is derived as:

$\begin{aligned} R_{CD}=\bar{r}_{LCP}^{*}\bar{r}_{LCP}-\bar{r}_{RCP}^{*}\bar{r}_{RCP}=2\left( r_{xx}^{R}r_{xy}^{I}-r_{yx}^{I}r_{yy}^{R} \right)\#\left( S6 \right) \end{aligned}$

where *r^*^* is the conjugate matrix of reflectivity, *r^R^* and *r^I^* represent the real and imaginary part of the reflectivity, respectively. The final expression of *R_CD_* is:

$\begin{aligned} R_{CD}=\frac{4\xi(\frac{\gamma_{ay}}{\gamma_{ry}}-\frac{\gamma_{ax}}{\gamma_{rx}})({\gamma_{rx}\gamma_{ry})}^{\frac{3}{2}}}{\left| \xi^{2}+\left[ j\left( \omega_{0}-\omega_{x} \right)+\gamma_{rx}+\gamma_{ax} \right]\left[ j\left( \omega_{0}-\omega_{y} \right)+\gamma_{ry}+\gamma_{ay} \right] \right|^{2}}\#\left( S7 \right) \end{aligned}$

For each parameters, we have simulated the *△A* with different structures, and numerically fitted the *△A* to the *R_CD_* curve. For the change of *△Y*, as the shape and thicknesses of nanorods are the same, we assume that the absorption losses for each modes are identical ($\gamma_{a}=\gamma_{ax}=\gamma_{ay}$). The extracted parameter for each structure is shown in Fig. S2.

**Supplementary Note 2: Parameter analysis for structural CD**

Considering the resonance frequency of two modes collides, the $R_{CD}$ can be expressed as:

$\begin{aligned} R_{CD}=\frac{4\xi({\gamma_{rx}\gamma_{ry})}^{\frac{3}{2}}(\frac{\gamma_{ay}}{\gamma_{ry}}-\frac{\gamma_{ax}}{\gamma_{rx}})}{\left| \xi^{2}+\left( \gamma_{rx}+\gamma_{ax} \right)\left( \gamma_{ry}+\gamma_{ay} \right) \right|^{2}}\#\left( S8 \right) \end{aligned}$

We assume that the chiral metamaterials have been optimized and the losses are all constant. Next, we place a chiral molecule onto the chiral metamaterials. Such act will affect the chiral interaction between each mode, which induce a perturbation term $\Delta\xi$ to the equation. Therefore, the difference of $R_{CD}$ induced by the molecules can be written as:

$\begin{aligned} \Delta R_{CD}=R_{CD}\left( \xi+\Delta\xi\right)-R_{CD}\left( \xi\right)\#\left( S9 \right) \end{aligned}$

Considering the perturbation is too small to affect the whole the value of CD, we could further obtain where the perturbation induces larger difference by taking the derivative of $R_{CD}$ and solve the extremum value, which is:

$\begin{aligned} \lim_{\Delta\xi\to0} \frac{\Delta R_{CD}}{\Delta\xi}=\frac{dR_{CD}\left( \xi\right)}{d\xi}=\frac{4({\gamma_{rx}\gamma_{ry})}^{\frac{3}{2}}(\frac{\gamma_{ay}}{\gamma_{ry}}-\frac{\gamma_{ax}}{\gamma_{rx}})[(\gamma_{rx}+\gamma_{ax})(\gamma_{ry}+\gamma_{ay})-3\xi^{2}]}{\left| \xi^{2}+\left( \gamma_{rx}+\gamma_{ax} \right)\left( \gamma_{ry}+\gamma_{ay} \right) \right|^{3}}\#\left( S10 \right) \end{aligned}$

Hence, the extremum near field coupling coefficient can be solved as:

$\begin{aligned} \xi_{0}=\pm\sqrt{\frac{\left( \gamma_{rx}+\gamma_{ax} \right)\left( \gamma_{ry}+\gamma_{ay} \right)}{3}}\#\left( S11 \right) \end{aligned}$

Varying the loss parameters, we could observe the extremum value occurring, shown in Fig. S3(A). Changing the parameters of the losses, the local maximum value is also tuned, shown from Fig. S3(B) to S3(E). To further explore the influence brought by the losses, we assume the near field coupling has reached the extremum value $\xi_{0}$, then the maximum $R_{CD}$ can be written as:

$$\begin{aligned} R_{CD}\left( \xi_{0} \right)=\pm\frac{3\sqrt{3}}{4}\left[ \left( 1+\frac{\gamma_{ax}}{\gamma_{rx}} \right)\left( 1+\frac{\gamma_{ay}}{\gamma_{ry}} \right) \right]^{-\frac{3}{2}}\times\left( \frac{\gamma_{ay}}{\gamma_{ry}}-\frac{\gamma_{ax}}{\gamma_{rx}} \right)\#\left( S12 \right) \end{aligned}$$

We mapped the $R_{CD}$ value with different loss ratios, the results are shown in Fig. S3(F). When the loss ratio $\frac{\gamma_{ax}}{\gamma_{rx}}=\frac{\gamma_{ay}}{\gamma_{ry}}$, $R_{CD}$ is zero. If the two values have larger difference, $R_{CD}$ also becomes larger. Furthermore, when one of the loss ratios is zero, another will achieve an optimized value around 2. This result indicates an extreme value for *△R* ^1,2^ But this cannot happen for our system as the absorption loss should be a nonzero term for the plasmonic structures.

**Supplementary Note 3: Influence by metamaterial length and gap**

We have experimentally validated the influence brought by the length *L* and the gap *g* of nanorod structure, as shown in Fig. S5(A). We measured the CD and IR spectra with the varied lengths from 1.7 μm to 1.9 μm and varied gap from 100 nm to 200 nm. The results for CD spectrum are shown in Fig. S5(B) and S5(C). When the length increases, the resonant wavelengths of the plasmonic mode shift to longer wavelengths. Besides, the *△A* also decreases, which is because of the bandwidth limitation of our 1/4 waveplate (λ_0_=6 μm, *△*λ=400 nm). For the gap difference, 100 nm gap could provide a larger *△A* as smaller gap could provide larger field enhancement. The results for IR present similar performance as the CD spectrum, as shown in Fig. S5(D) and S5(E). Furthermore, we extracted the resonant wavelengths and the Q factor for both spectra and plotted with the length *L* and the gap *g*, as shown in Fig. S5(F). The Q factor is defined as:

$\begin{aligned} Qfactor=\frac{\lambda_{0}}{\Delta\lambda}\#\left( S13 \right) \end{aligned}$

where *λ_0_* is the center wavelength for the resonance peak, and *△*λ is the FWHM bandwidth. The wavelength shift is almost linear as the nanorod length changes, indicating our method of designing metamaterial is applicable for the whole mid-IR ranges. Besides, although there is little difference of the Q factor as gap changes for CD spectrum, a higher Q factor is observed with smaller gap for IR spectrum. Therefore, smaller nanogap is still necessary for chiral metamaterial design.

We have also compared the sensing performance between 100 nm gap and 200 nm gap, as shown from Fig. S8(A) to Fig. S8(D), where 100 nm gap has larger CD and can perform larger VCD enhancement for BSA sensing. Besides, we have also tried the fabrication of sub-100 nm gaps, as shown in Fig. S8(E). This indicates the potential of fabricating structures with smaller fingerprint using the same platform. Especially, larger field enhancement can be experienced by the molecules if they are introduced into the gap regions. However, in our design, sub-100 nm gaps may not promise better performance. One of the possible issues is the strong coupling induced Rabi-splitting, as shown in Fig. S8(F). We have used FDTD method (Lumerical FDTD) to simulate the device with smaller gaps. The resonant peak is split into two when reducing the gap lengths under the linear polarization. Such resonance splitting is known as Rabi-splitting, which is induced by the strong coupling when two nanorods are close. We further simulate the R_CD_ with smaller gaps, and find that the resonance splitting still exists, as shown in Fig. S8(G). However, the R_CD_ value is also decreased. Such phenomenon is interesting, but not within the scope of our work. One potential application for such nanogap is to create a broadband sensing platform, as the range of CD enhancement is also broadened enabled by these split resonances.

**Supplementary Note 4: Parameter analysis for chiral sensing**

We have discussed the parameter influences by the losses ($\frac{\gamma_{ay}}{\gamma_{ry}}-\frac{\gamma_{ax}}{\gamma_{rx}}$) with extremum near field coupling coefficient $\xi_{0}$. We will further discuss the influences when the coupling coefficient is varied. Similarly, we assume that the near field coupling would have a perturbation when molecules drop onto the sensing area. Therefore, the perturbation will induce a slight $R_{CD}$ shift, while the derivative of $R_{CD}$, denoted as $R_{CD}'$, represents for the changing rate of $R_{CD}$. The physical meaning of $R_{CD}'$ is the sensitivity of CD spectrum when near field coefficient is differed with a small value. The plotted results for $R_{CD}$ and $R_{CD}'$ are shown in Fig. S6(A). Noted that when $\xi$ is close to zero, a largest $R_{CD}'$ is achieved, which refers to weak near field coupling between two resonant modes. We further use the previously extracted absorption and radiation losses from C+3 to C-3 structures and calculate the $R_{CD}'$ as a function of $\xi$, as shown in Fig. S6(B). It can be observed that C+3 and C-3 structures have larger $R_{CD}'$ than C-2 and C+2 structures, followed by C-1, C+1, and C0 structures. Such result agrees well with the circular dichroism distribution shown in Fig. 2E. However, although weak near field coupling regime could provide larger sensitivity of the $R_{CD}$, the signal needs amplifying by superchiral field before it could be observed by the CD spectrum. We have further calculated and plotted the enhancement by superchiral field and the related $R_{CD}'$ for each structure. The $R_{CD}'$ for each structure is determined according to the extracted $\xi$ shown in Fig. 2C(ii). The results are shown in Fig. S6(C). Although the C0 structure could provide larger $R_{CD}'$, the superchiral field enhancement is near zero. Therefore, we multiply these two factors for each structure, shown in Fig. S6(D). The expression is written as $R_{CD}'\cdot\frac{\bar{\Delta C}}{U\cdot C_{0}}$. To understand in a more intuitive way, the former term determines the sensitivity to the signal perturbation (such as molecules), while the latter term determines the amplification to the detected signal, which is the superchiral field experienced by the molecules. This platform applies for our structures, as the value decreased from the maximum to the minimum when varied from C+3 to C-3. Such framework could supervise the design and optimization process for chiral metamaterial sensors.

**Supplementary Note 5: Sensing characterization at different volumes and noise measurement of optical testing setup**

To characterize the minimum detection volume for our sensing platform, we have measured the IR sensing results for BSA at the concentration of 12.5 ng/μL with sample volume of 2 μL and 1 μL, respectively. The results are shown in Fig. S9(A). It can be noticed that the *△R* signal for 2 μL is larger than 1 μL at the absorption peak of 1650 cm^-1^, which agrees with the sample volume. Although we could distinguish the volume change in this way, the signal fluctuation is observable at other wavenumbers, which is brought by the whole measurement setup. The reason of this kind of noise is mainly from the interference-like pattern, as shown in Fig. S9(B). As we have mentioned in the optical characterization part, we used Spero^@^ Chemical Imaging Microscope for the whole measurement, which contains a QCL laser as mid-IR light source. Therefore, this noise is mainly from the laser’s high degree of coherence, which leads to interference-like pattern ^3^. We have repeated testing the VCD spectra on these areas, the results are shown in Fig. S9(C), indicating that the noise level is around 0.01. This may influence the VCD signal when the concentration is lower. One possible method to mitigate such coherence-induced interference pattern is to substitute the QCL setup with a classical FT-IR.

**Supplementary Note 6: Demonstration of enantioselective sensing**

In the manuscript, we have demonstrated the enhanced VCD sensing of protein secondary structures, which proves the potential of biomedical and clinical diagnosis applications. For stereochemistry related applications, we have also demonstrated using enantiomers, which have the same connectivity but opposite 3D shapes. The molecules used are D-glucose (Sigma-Aldrich, product G8210) and L-glucose (Sigma-Aldrich, product G5500). We fabricate several new chiral metamaterials with longer resonance wavelength to match the vibrational mode of glucose, ranging from 1000 cm^-1^ to 1400 cm^-1^ ^4^. The glucose was dissolved in DI water and formed a solution with a concentration of 2 mg/ml. For each measurement, the glucose solution was fetched using a micropipette (~2 μl) and dropped on to the metamaterials.

The fabricated devices are shown in Fig. S17(A). The length of the nanorods is 3.5 μm, which corresponds to a resonance wavenumber near 1100 cm^-1^. The enhanced IR sensing performance of D-glucose and L-glucose are shown in Fig. S17(B). Unfortunately, we didn’t measure any obvious vibration transitions near 1100 cm^-1^, but an absorption peak can still be observed near 1225 cm^-1^. We have simulated the near field enhancement at these two wavelengths, and find that both wavelength present field enhancement, while the resonance peak near 1225 cm^-1^ shows smaller enhancement factor (around 245), which is the reason why the vibration peaks of glucose can still be enhanced. Furthermore, we used a 1/4 waveplate with a centre wavelength of 8500 nm to measure the VCD enhancement of the absorption peak. The results are shown in Fig. S17(C) and Fig. S17(D), where the VCD signal of L-glucose is amplified when coated on C+3 metamaterials, and the D-glucose signal is enhanced using C-3 metamaterials. Compared with the VCD signal of D-glucose without any enhancement (~10^-5^)^4^, our results show an enhancement of VCD signal with at least 3 orders of magnitudes.

In summary, we successfully demonstrate the enhanced VCD sensing of glucose enantiomers. Although for the current results, the vibrational mode has not matched the resonance peak of metamaterials, we can still observe an enhanced VCD signal around 3 orders of magnitudes. In future, we will do extensive survey for the resonance peak matching process, will may provide larger enhancement factor for glucose molecules sensing.


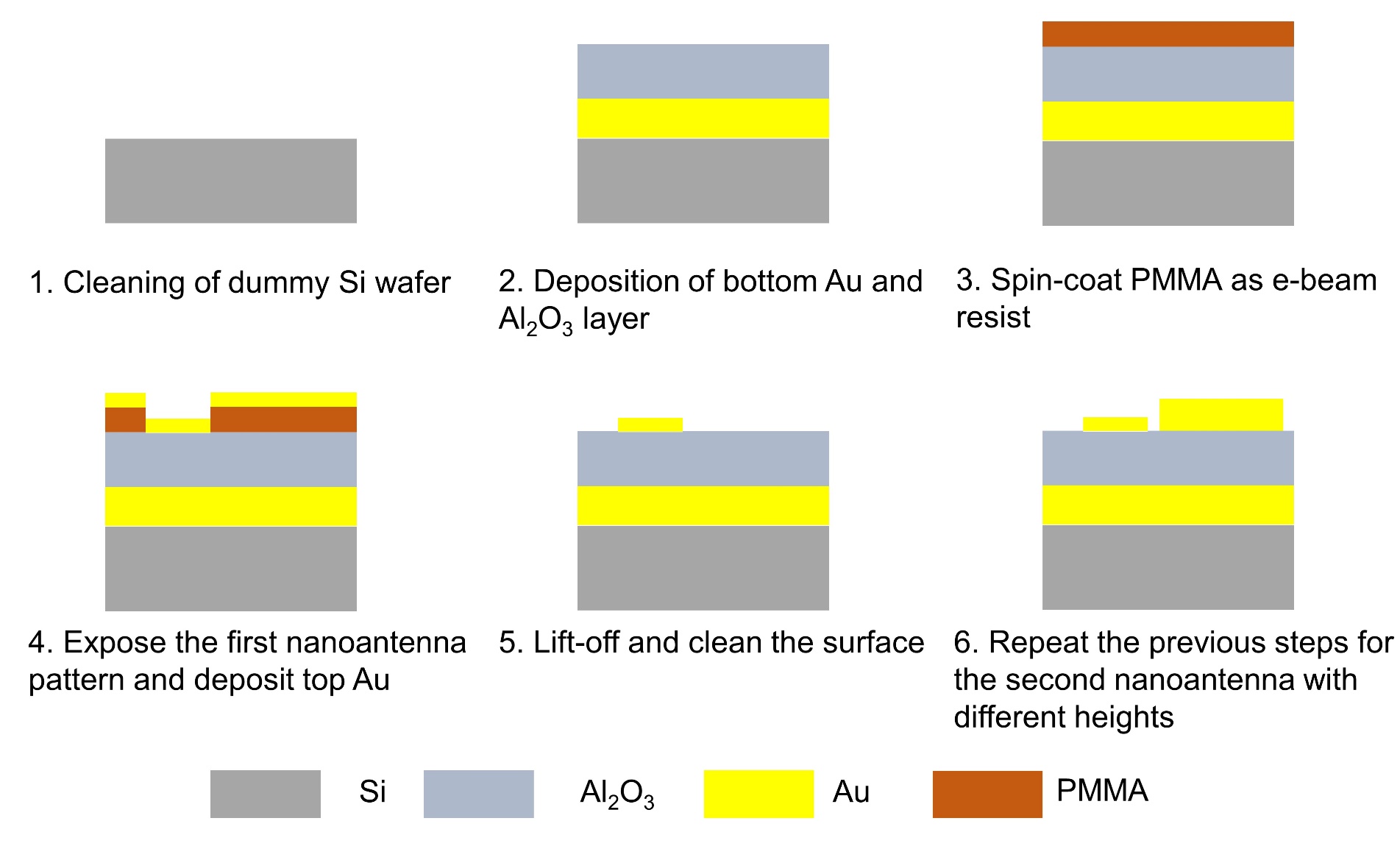


**Fig. S1: Fabrication process of double-rod chiral metamaterials.**


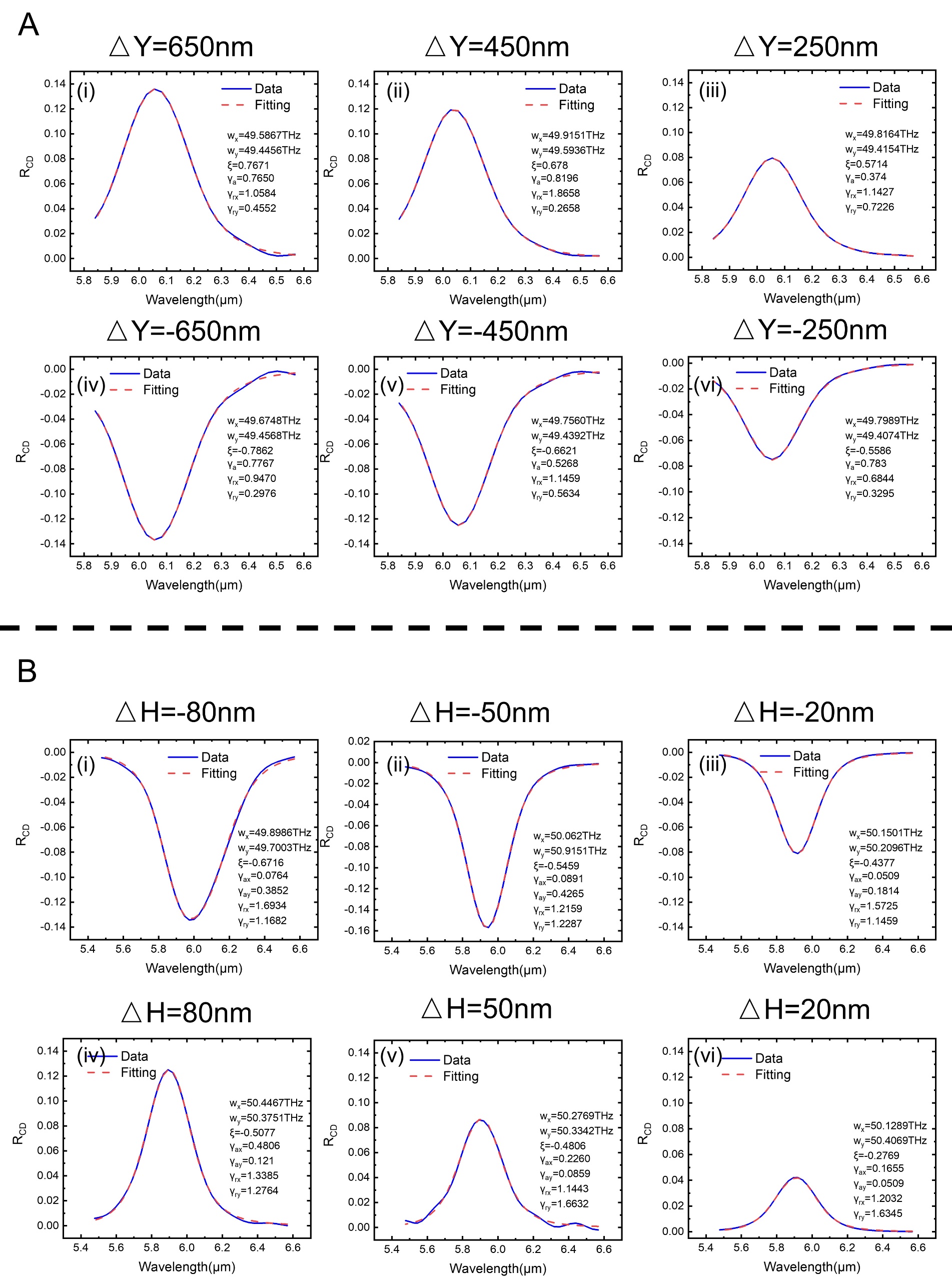


**Fig. S2:** **Parameter extraction for chiral metamaterials.** (**A**) Parameter extraction of *△Y* from 650 nm to -650 nm. (**B**) Parameter extraction of *△H* from -80 nm to 80 nm.


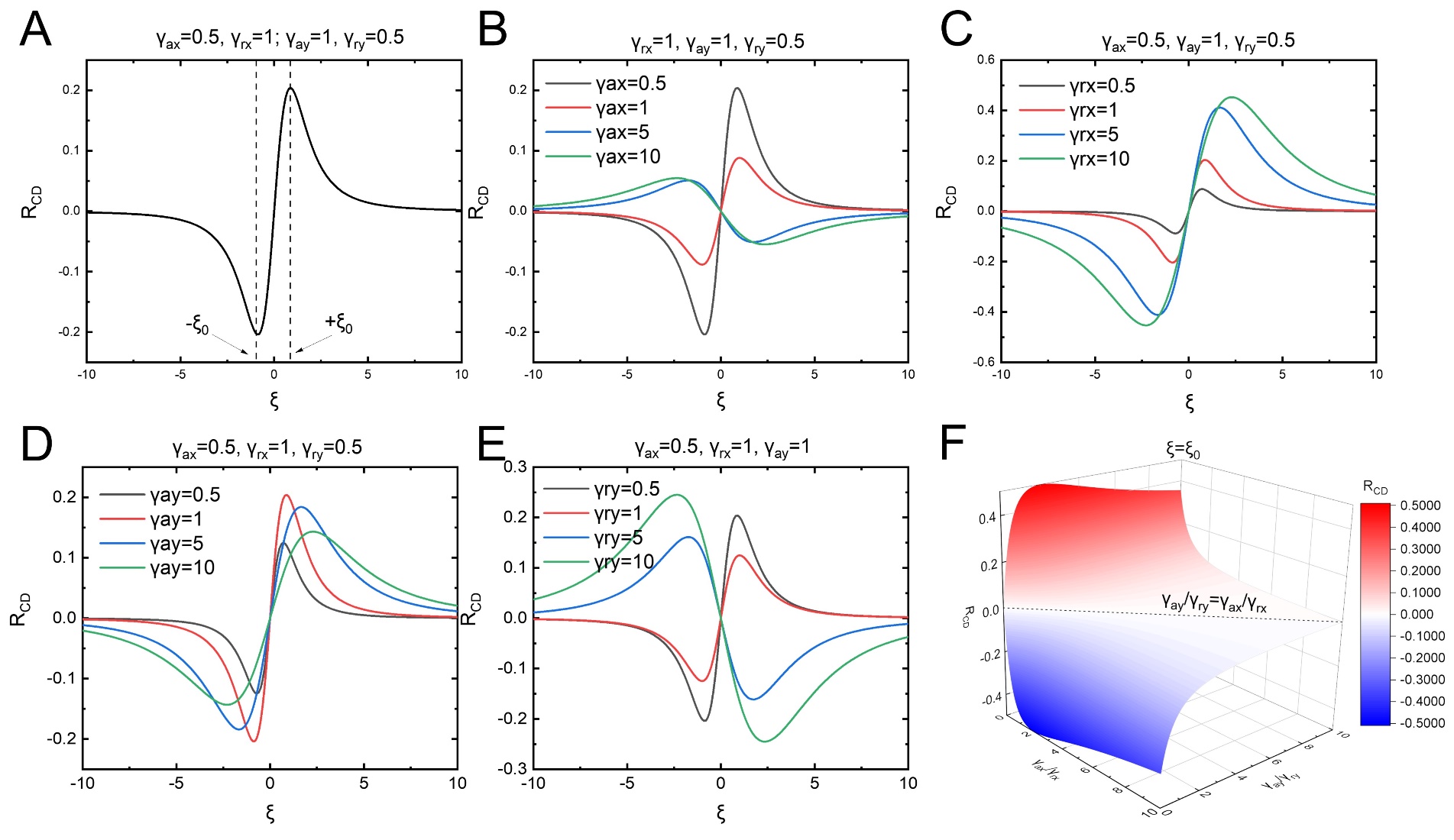


**Fig. S3:** **Parameter analysis for TCMT model.** (**A**) Calculated $R_{CD}$ with varied near field coupling coefficient $\xi$. Two global extremum value are noted as $\xi_{0}$ and ${-\xi}_{0}$. (**B** to **E**) Calculated $R_{CD}$ with varied losses. (**F**) Mapped $R_{CD}$ results at the extremum value ${\xi=\xi}_{0}$. The equal loss ratio $\frac{\gamma_{ax}}{\gamma_{rx}}=\frac{\gamma_{ay}}{\gamma_{ry}}$ could result in zero $R_{CD}$ value.

**
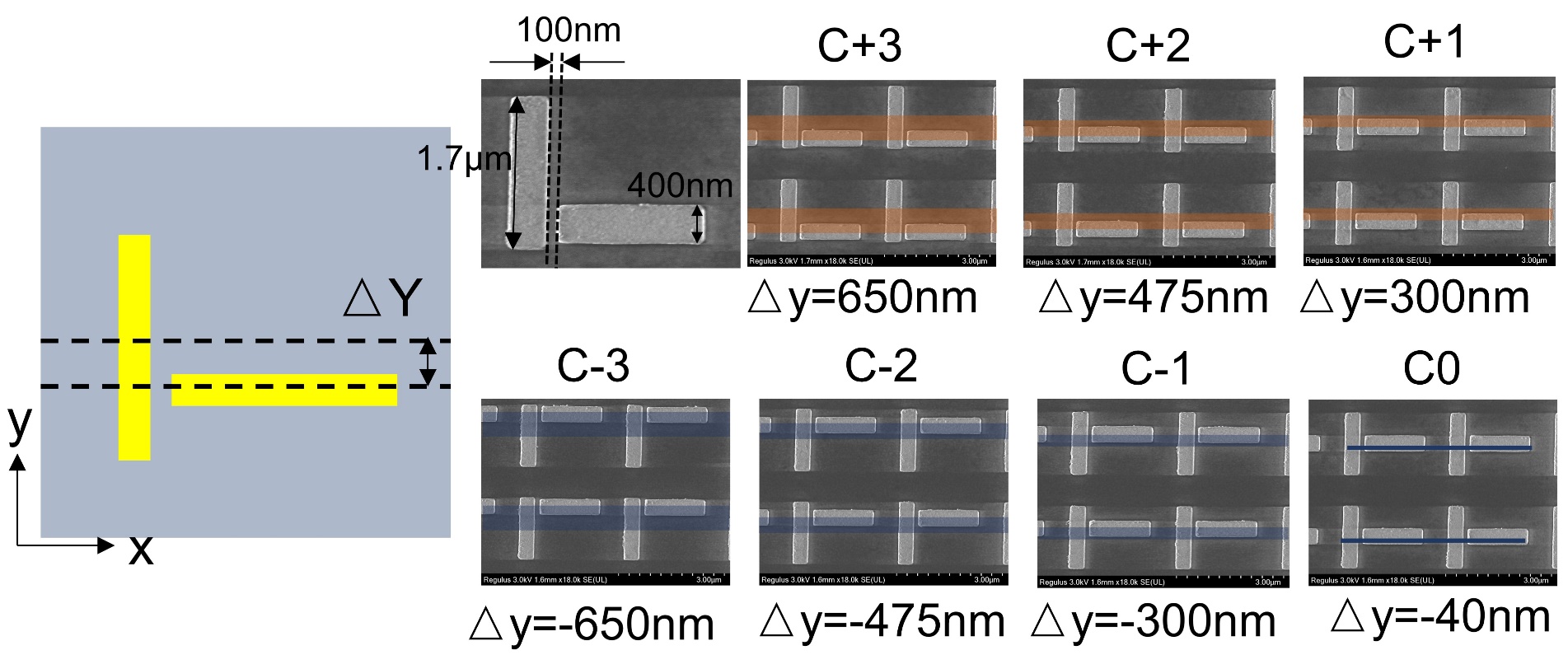
**

**Fig. S4:** **Device SEM image from C-3 to C+3**


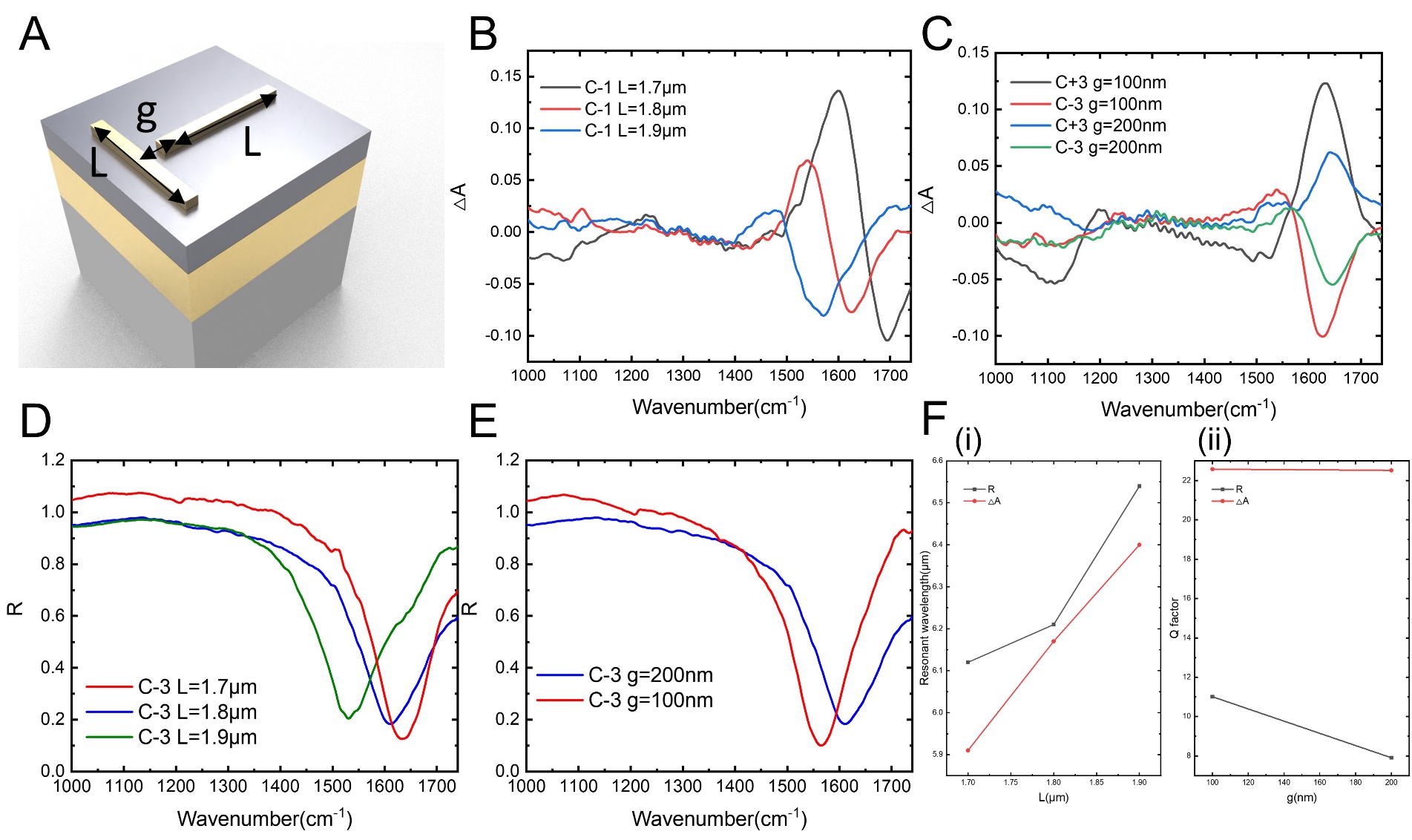


**Fig. S5:** **Device planar geometry characterization.** (**A**) Schematic showing of the tuned geometric parameter. *L* is the length for each single rod and *g* is the gap length between the double rods. (**B** to **C**) Measured *△A* with varied *L* and *g*. (**D** to **E**) Measured *R* with varied *L* and *g*. (**F**) Calculated wavelength shift with varied *L* (**i**) and calculated wavelength shift with varied *g* (**ii**).


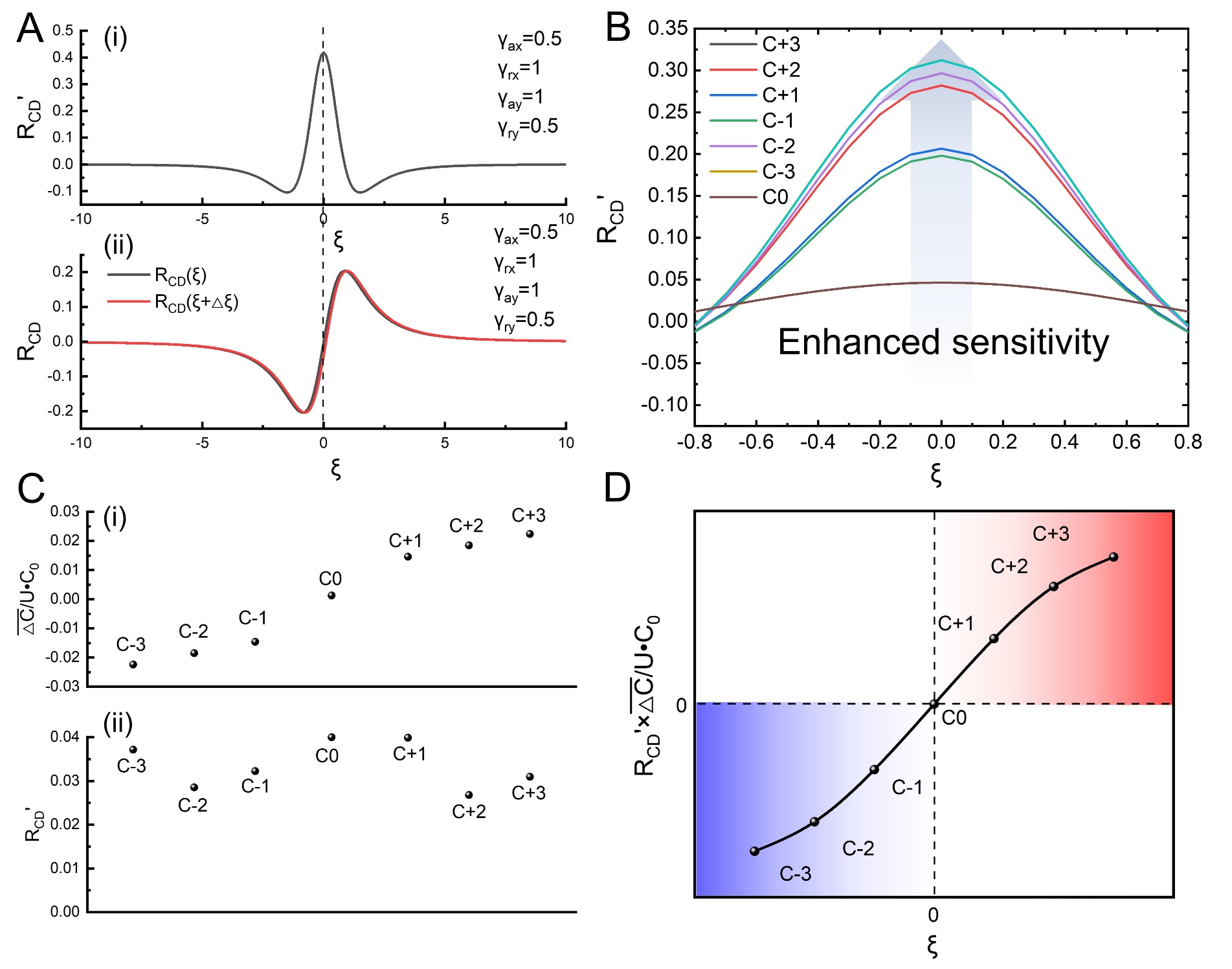


**Fig. S6:** **Parameter analysis for chiral sensing.** (**A**) Calculated $R_{CD}'$ (**i**) and $R_{CD}$ with varied $\xi$ (**ii**). The red curve in (**ii**) indicates the perturbation when $\xi\to\xi+\Delta\xi$. (**B**) Calculated $R_{CD}'$ with different devices with varied $\xi$. The loss and near field coupling coefficient for each device are extracted from curve fitting. (**C**) Superchiral field enhancement of each device (**i**) and $R_{CD}'$ of each device (**ii**). The $\xi$ for each device are selected according to previous curve fitting results. (**D**) Calculated $R_{CD}'$ multiple the surperchiral field enhancement with different $\xi$.


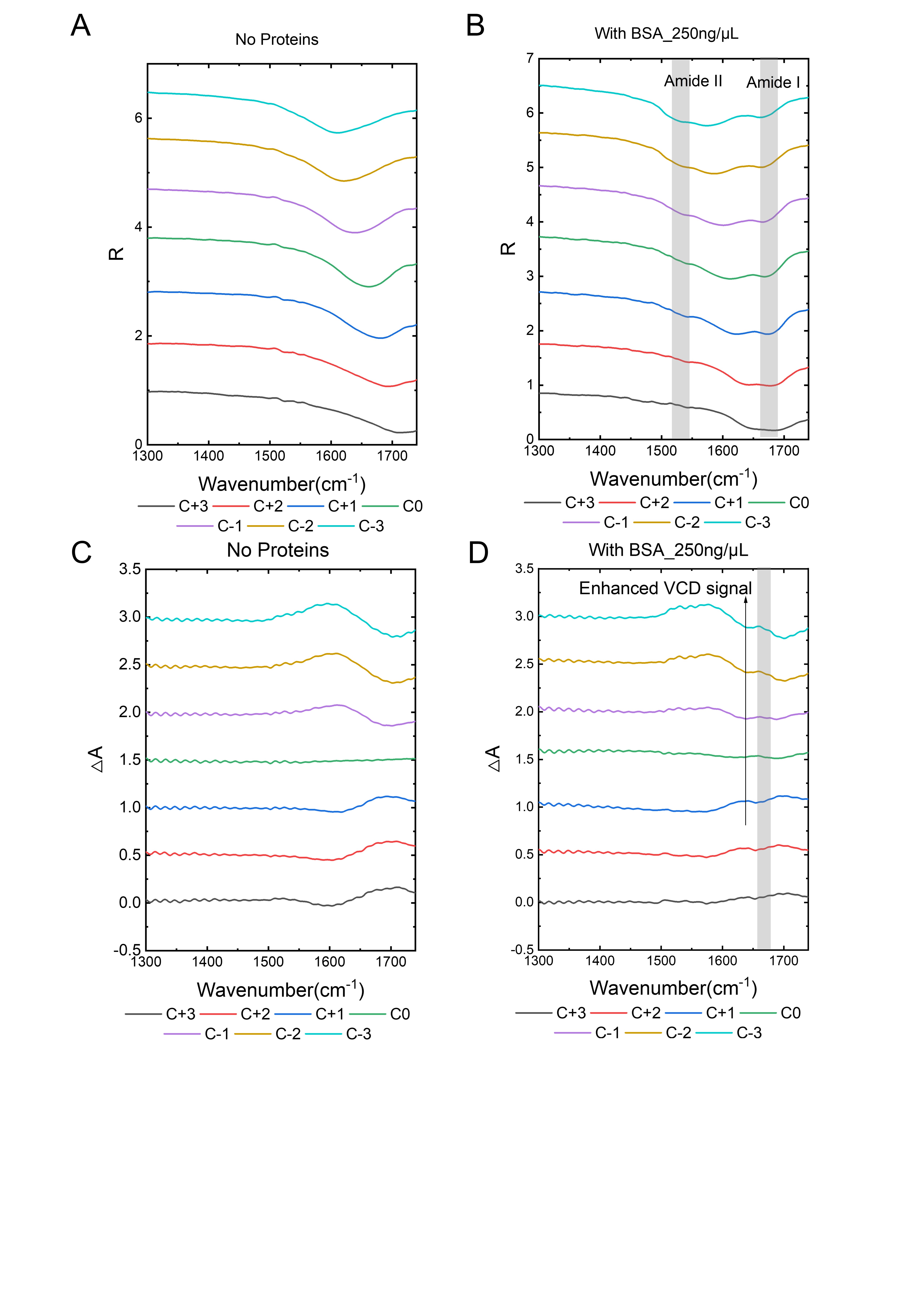


**Fig. S7:** **Sensing characterization from C-3 to C+3.** (**A** and **C**) Measured *R* and *△A* of devices from C+3 to C-3 without any molecules. (**B** and **D**) Measured *R* and *△A* of devices from C+3 to C-3 with BSA at concentration 250 ng/μL.


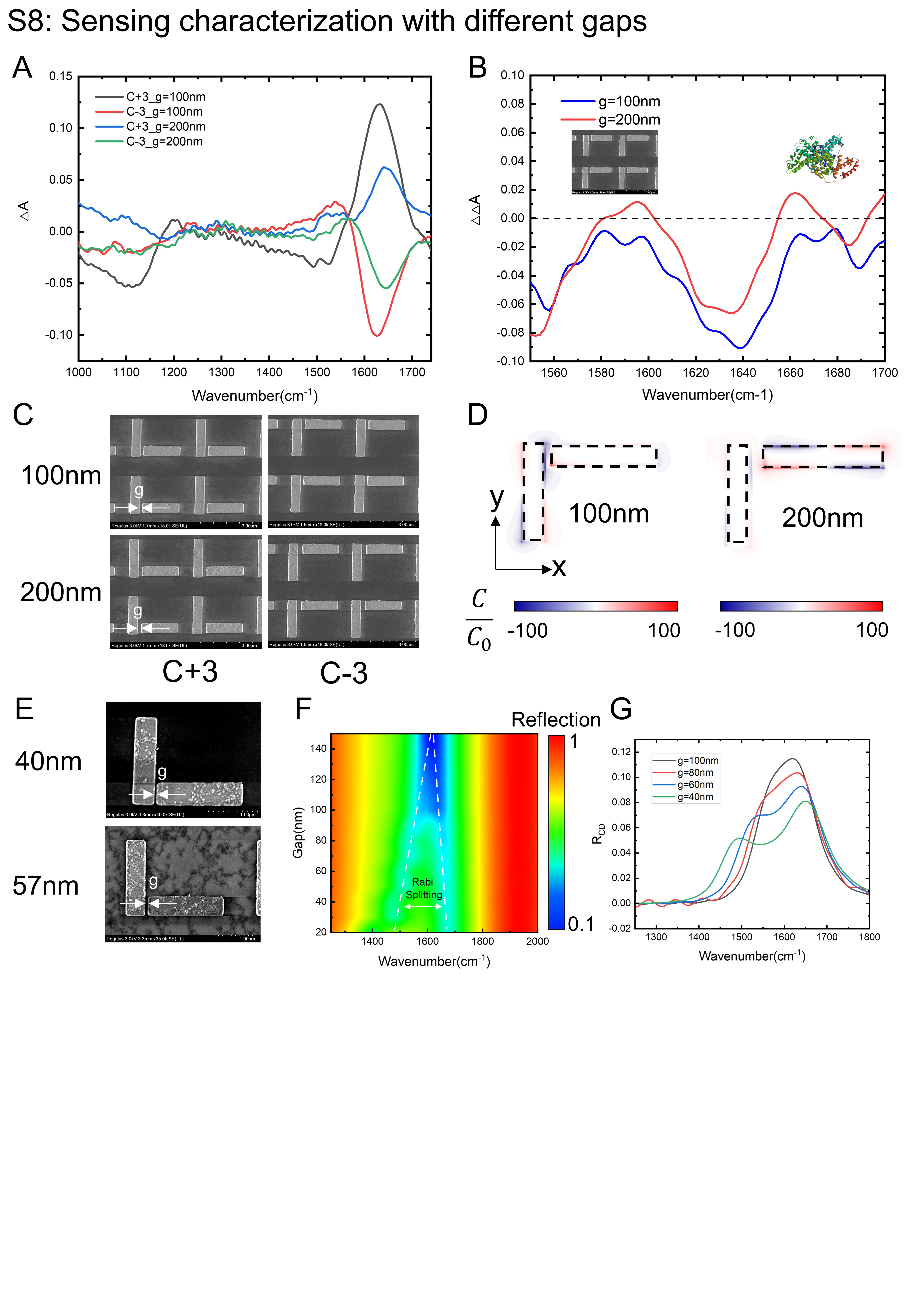


**Fig. S8:** **Sensing characterization with different gaps.** (**A**) Measured *△A* of devices C+3 and C-3 with varied gap lengths. (**B**) Measured *△△A* of devices C+3 and C-3 with varied gap lengths. (**C**) SEM images of devices C+3 and C-3 with varied gap lengths. (**D**) Optical chirality simulation of devices C+3 and C-3 with varied gap lengths. (**E**) Demonstration for fabricating sub-100 nm nanostructures. The 40 nm and 57 nm gaps indicate the possibility of developing nanosensors with sub-100 nm gap. (**F**) Simulated reflection spectra of metamaterial with reduced gap length. When the gap is smaller than 100 nm, the resonance peak is split into two, with higher reflection window. (**G**) Simulated reflection circular dichroism of metamaterial with varied gap length. The R_CD_ also presents resonance split with smaller gaps.


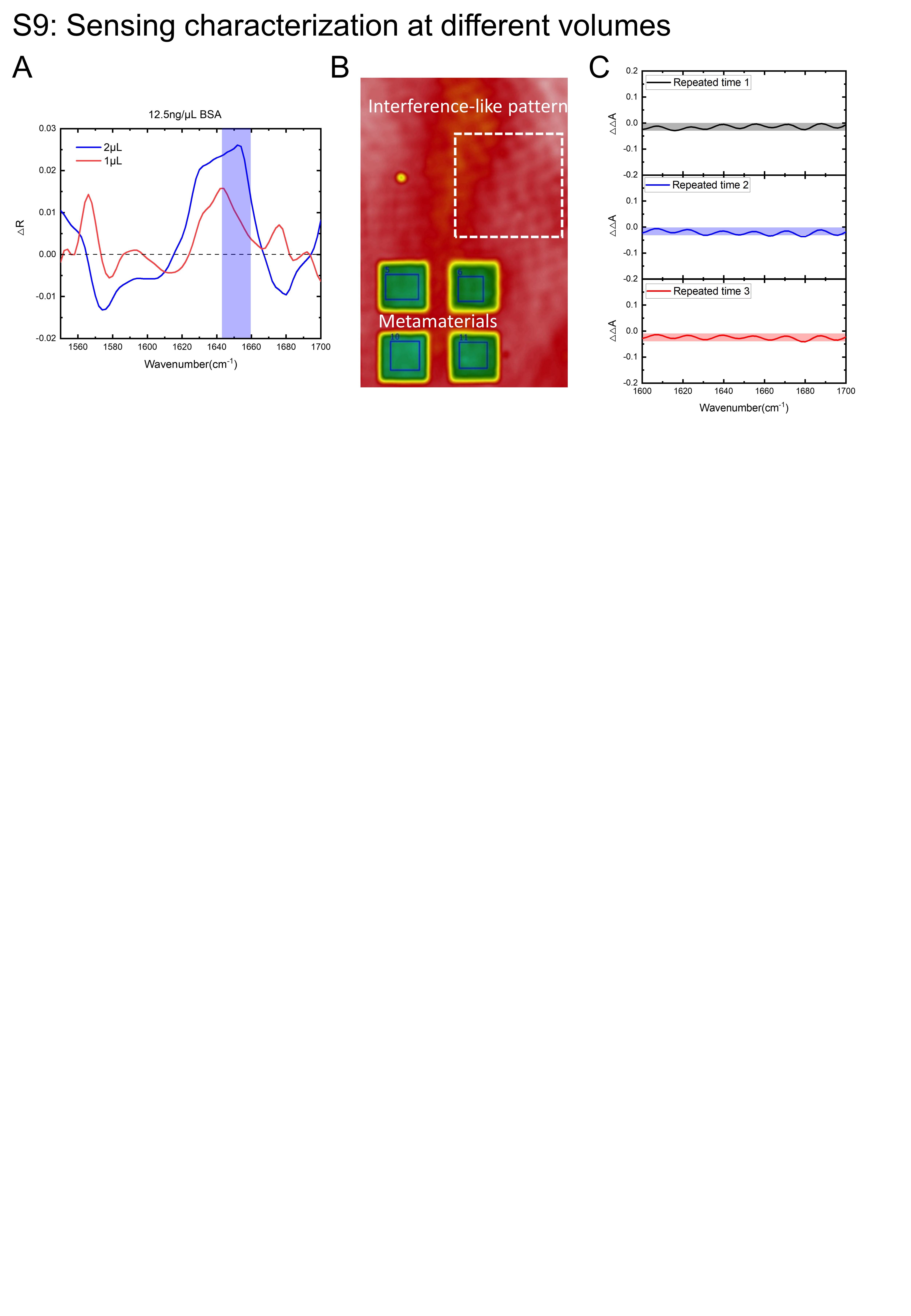


**Fig. S9:** **Sensing characterization with different volumes.** (**A**) IR absorption signal of 2 μL and 1 μL BSA solution with 12.5 ng/μL concentration. (**B**) IR camera image of the device. The red colour represents the Al_2_O_3_ substrate, while the green colour represents the metamaterials. (**C**) Experiment of the VCD signal on Al_2_O_3_ substrate. Three repeated tests were implemented to obtain the VCD curves.


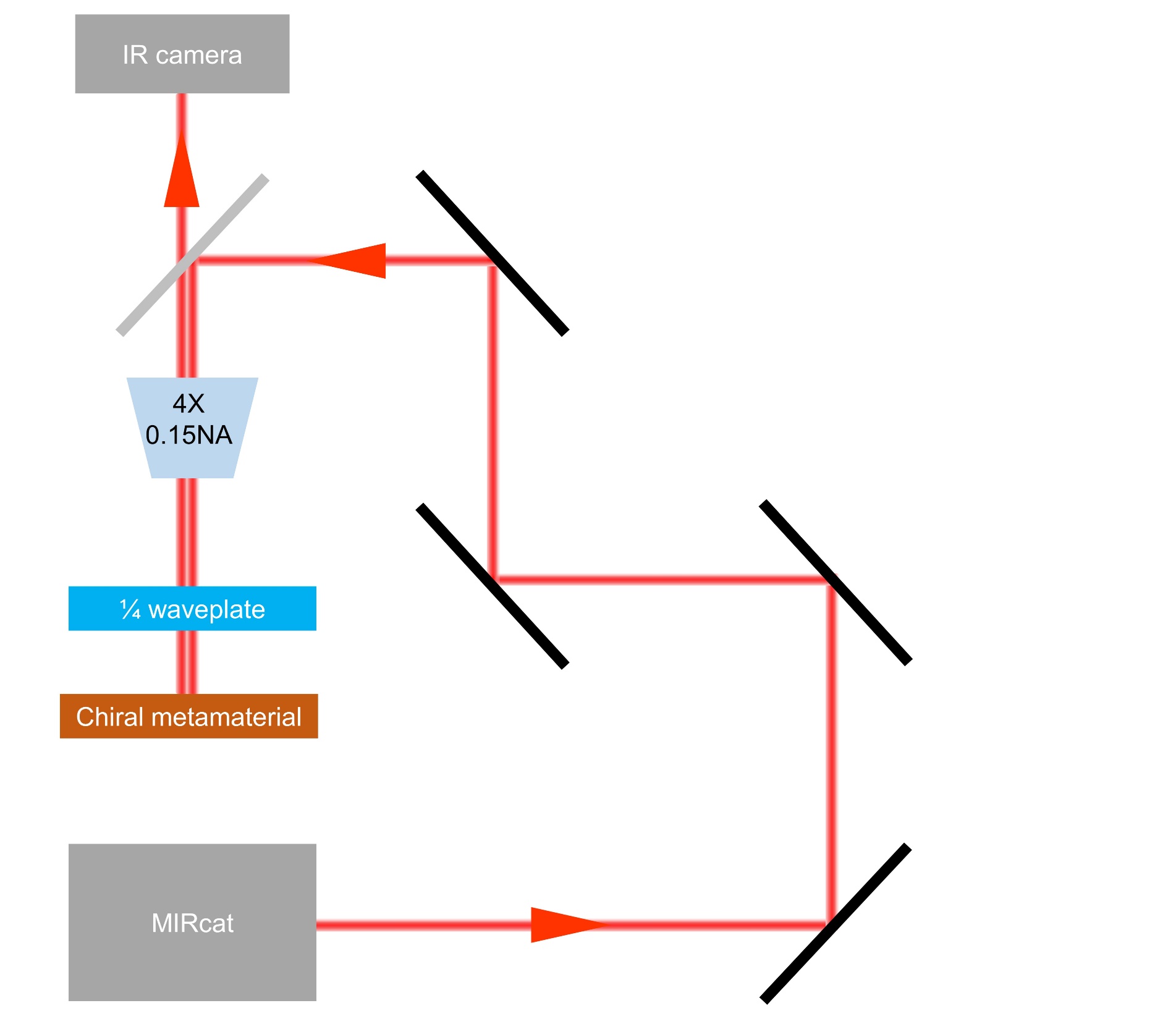


**Fig. S10:** **Optical measurement setup**


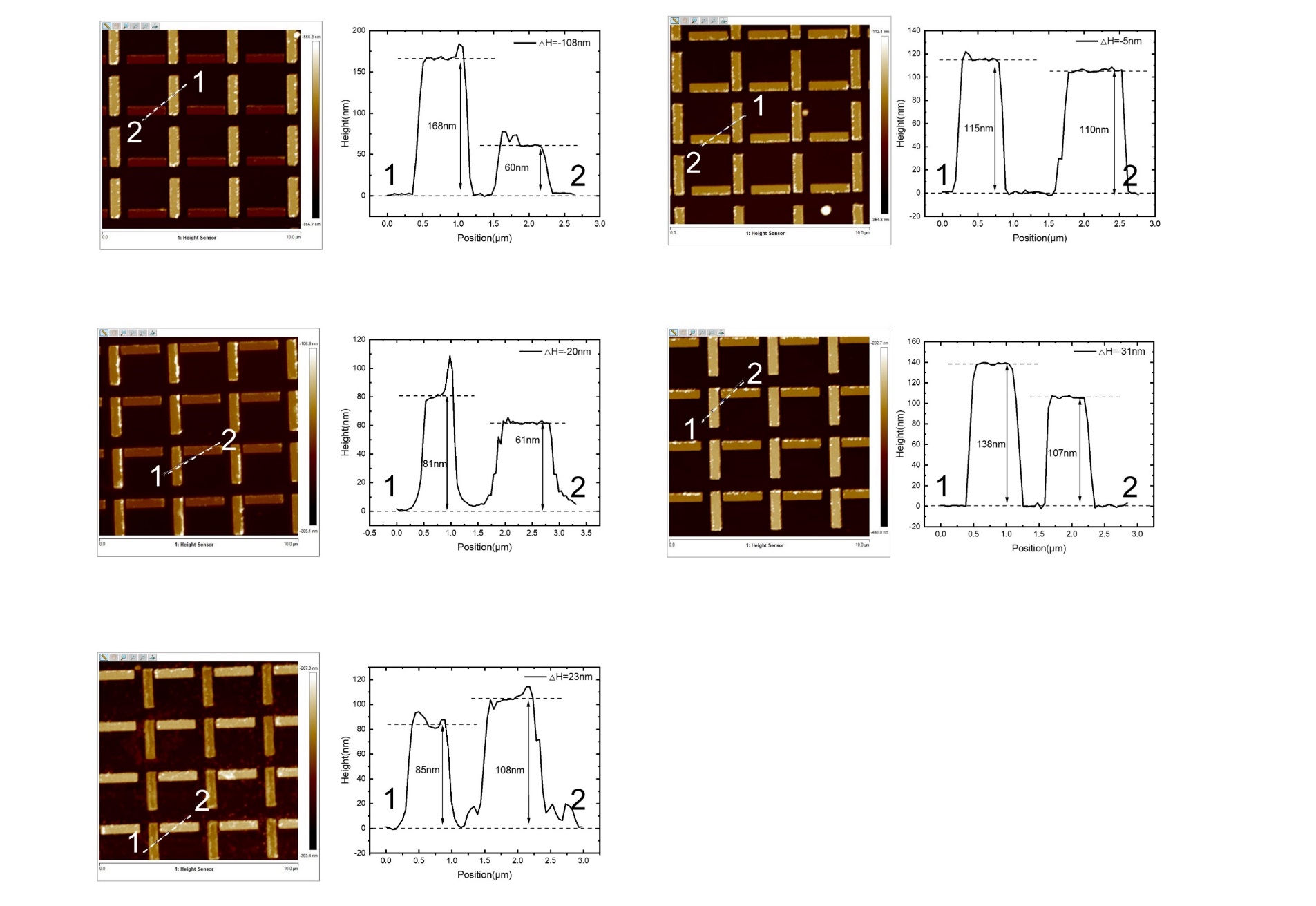


**Fig. S11:** **AFM characterization of the antenna thickness**


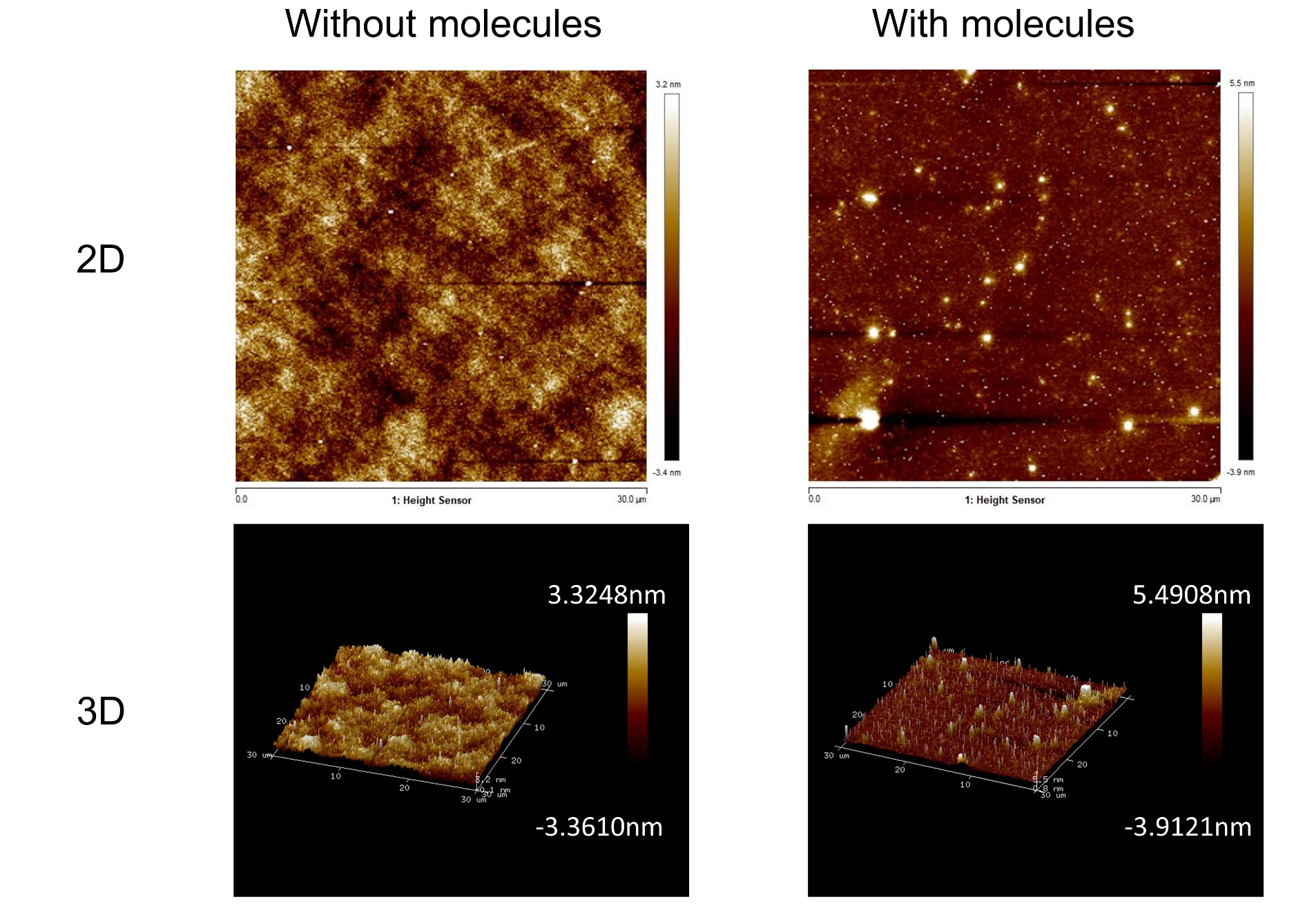


**Fig. S12:** **AFM characterization of the estimated molecule numbers.**

**
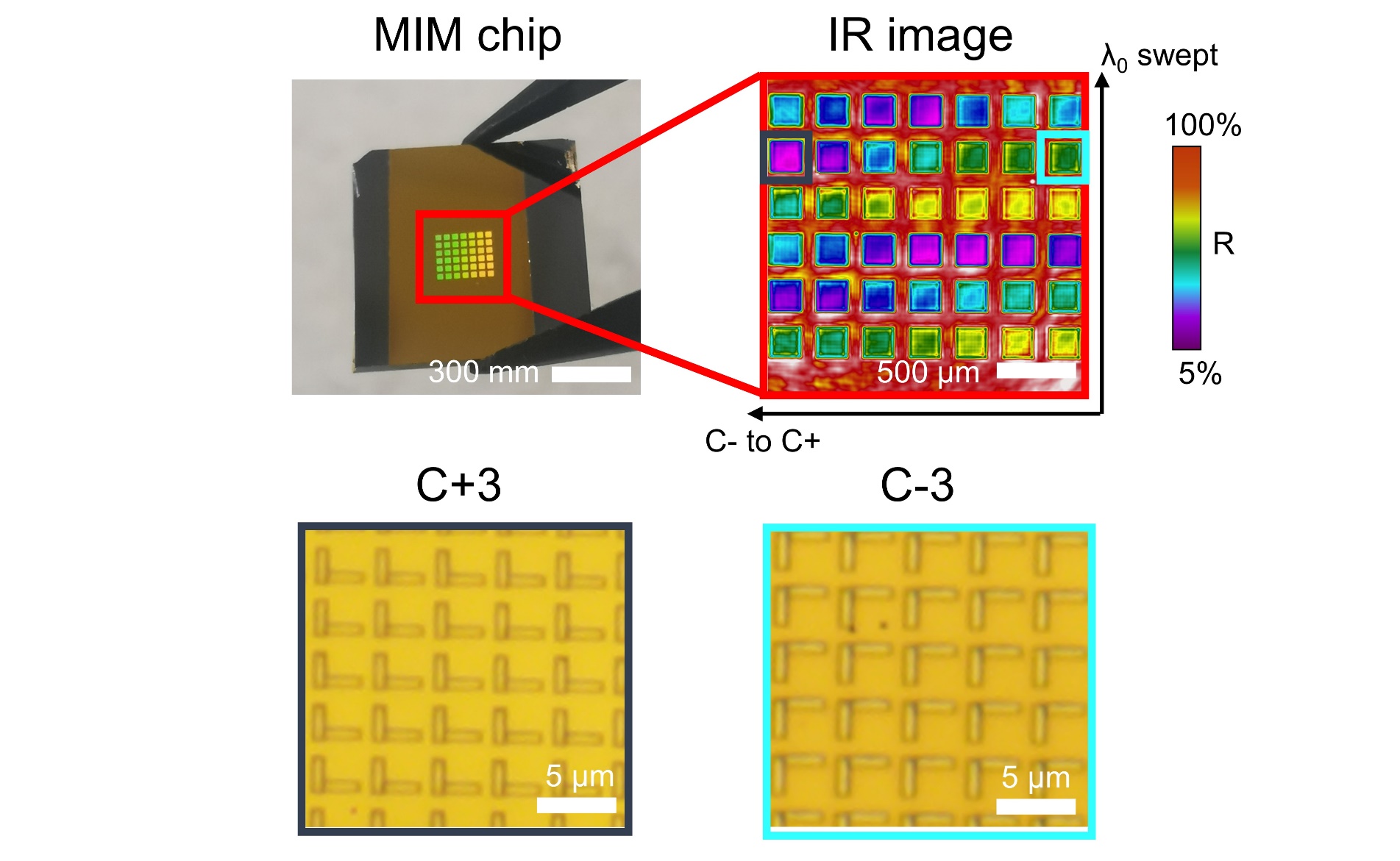
**

**Fig. S13: IR and OM image of the IRCPMs array for broadband sensing applications.** The fabricated IRCPMs array consists of a 7×6 metamaterial array. Each metamaterial region has a footprint of 200 × 200 μm^2^. Each column has different chirality varied from C+3 devices with positive optical chirality to C-3 devices with negative optical chirality, while the middle region is achiral C0 devices. Each row has varied resonant wavelengths, ranging from 1500 cm^-1^ to 1700 cm^-1^. As shown in the IR image under the incident light of 1650 cm^-1^, where different colors indicates the absorption difference of each metamaterial cells. Such IRCPMs array provides enhanced VCD sensing covering the full wavelength range of amide I and amide II vibrations for protein and peptide molecules.


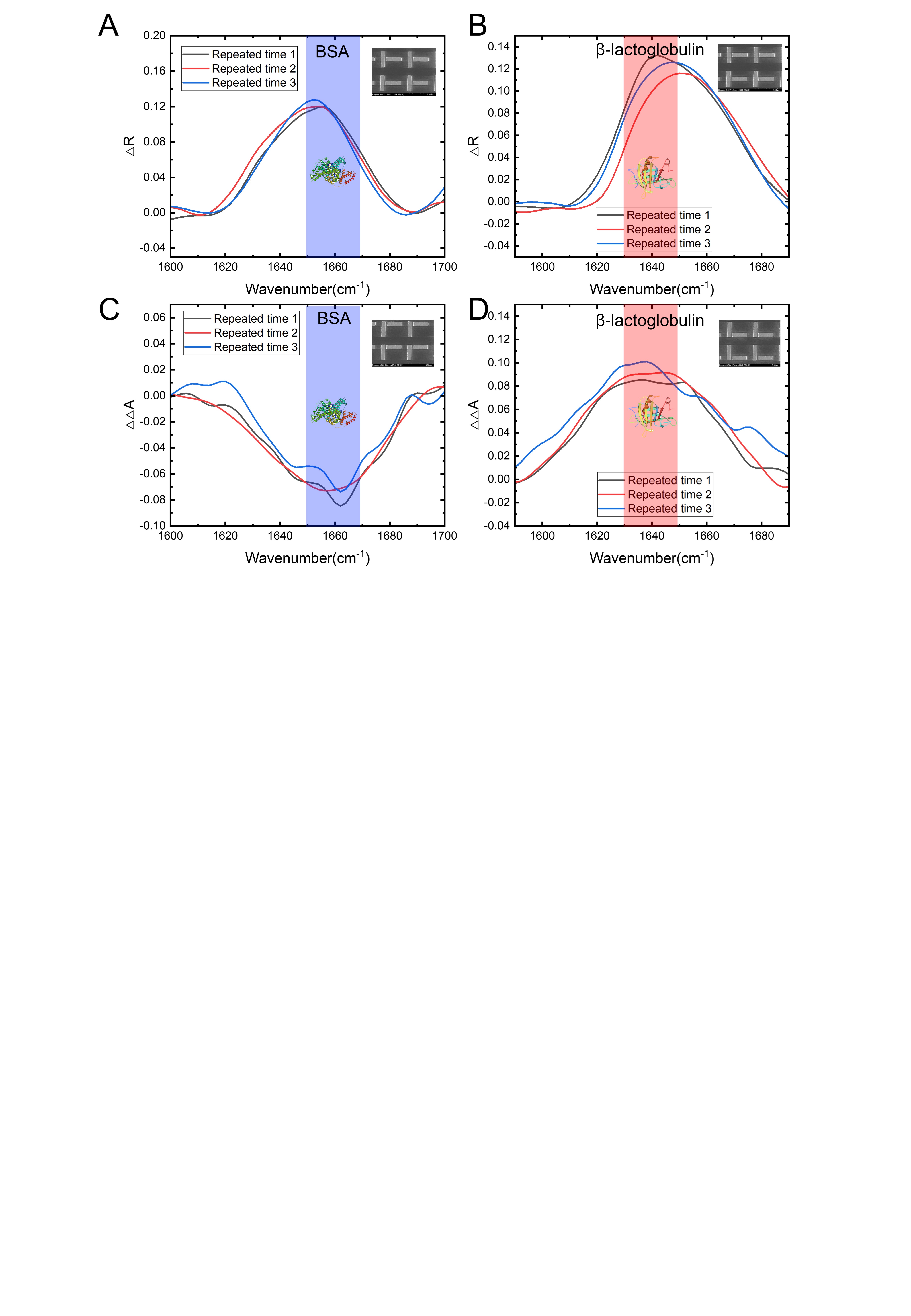


**Fig. S14: Repeated testing results of enhanced protein IR absorption and VCD signals.** (**A**) Enhanced IR absorption signal of BSA coated on C0 metamaterials. (**B**) Enhanced IR absorption signal of β-lactoglobulin coated on C0 metamaterials. (**C**) Enhanced VCD signal of BSA coated on C-3 metamaterials. (**D**) Enhanced VCD signal of β-lactoglobulin coated on C+3 metamaterials.

**
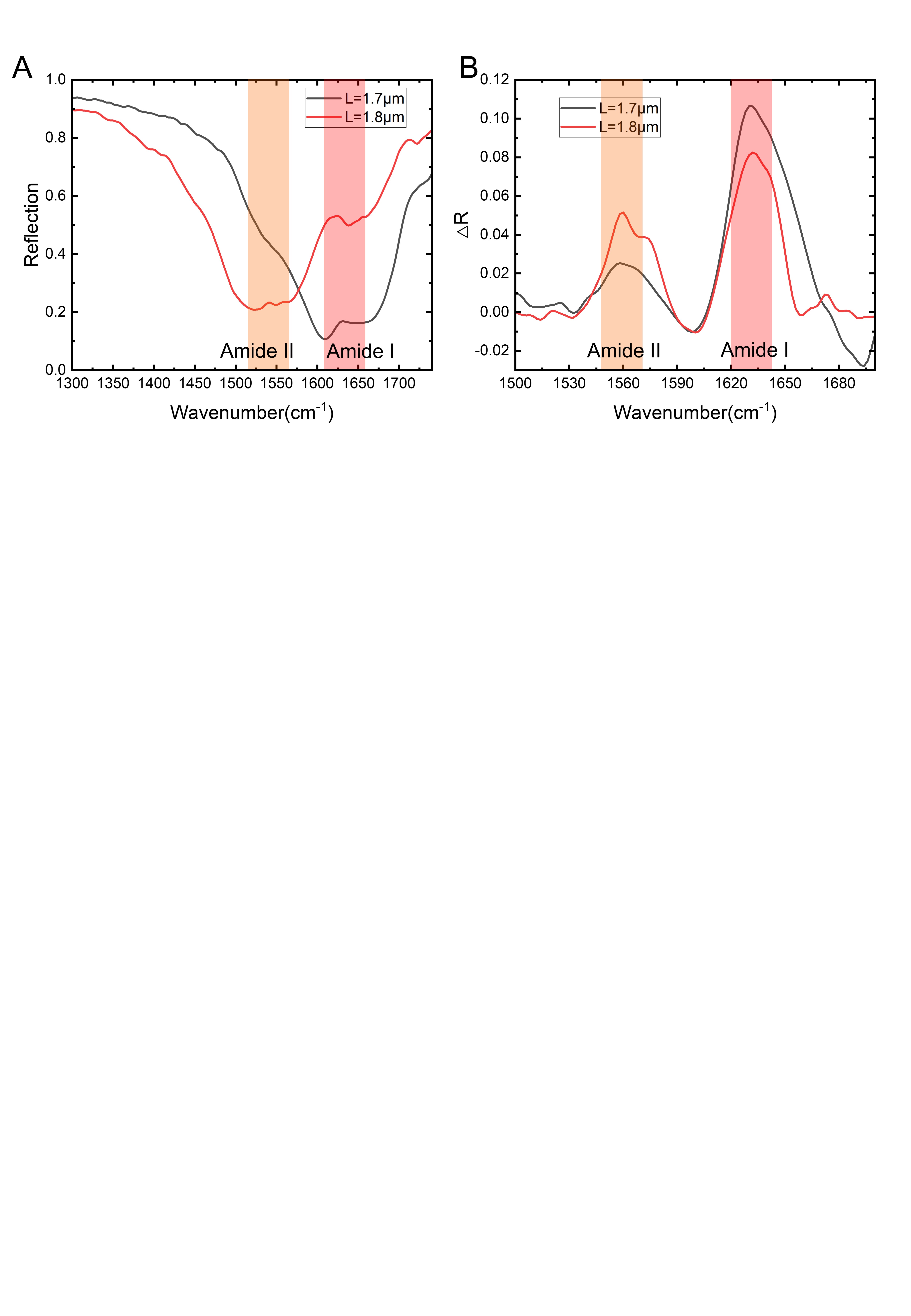
**

**Fig. S15: Characterization of β-lactoglobulin molecules using metamaterials with different nanorod lengths.** (**A**) Reflection spectrum of the C0 metamaterials coated with β-lactoglobulin molecules. The black and red curves represent different lengths of the nanorods. (**B**) Enhanced molecular IR signal after removing the background of metamaterials. The amide I and amide II peaks are enhanced accordingly when coated onto metamaterials with different lengths.

**
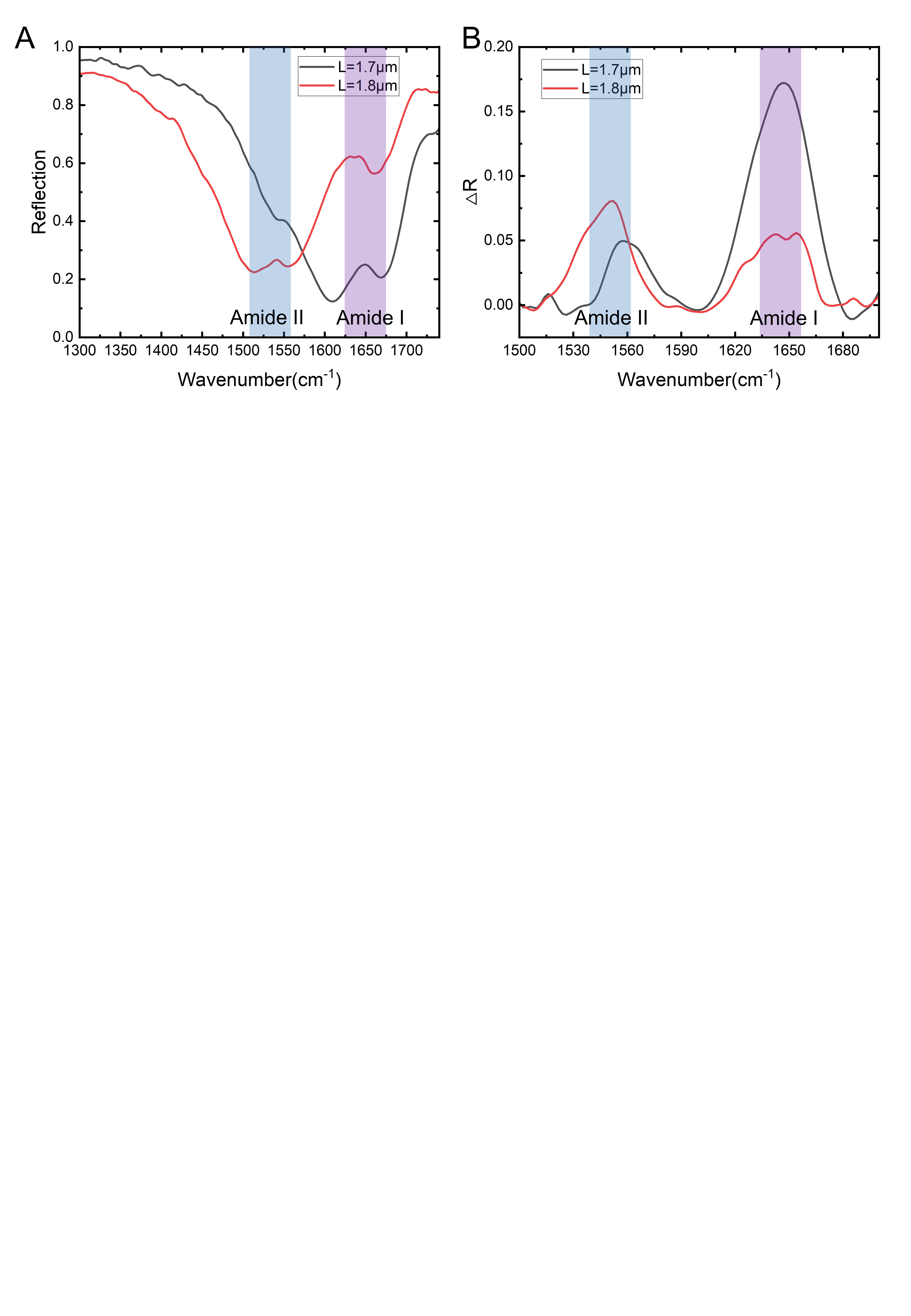
**

**Fig. S16: Characterization of BSA molecules using metamaterials with different nanorod lengths.** (**A**) Reflection spectrum of the C0 metamaterials coated with BSA molecules. The black and red curves represent different lengths of the nanorods. (**B**) Enhanced molecular IR signal after removing the background of metamaterials. The amide I and amide II peaks are enhanced accordingly when coated onto metamaterials with different lengths.

**
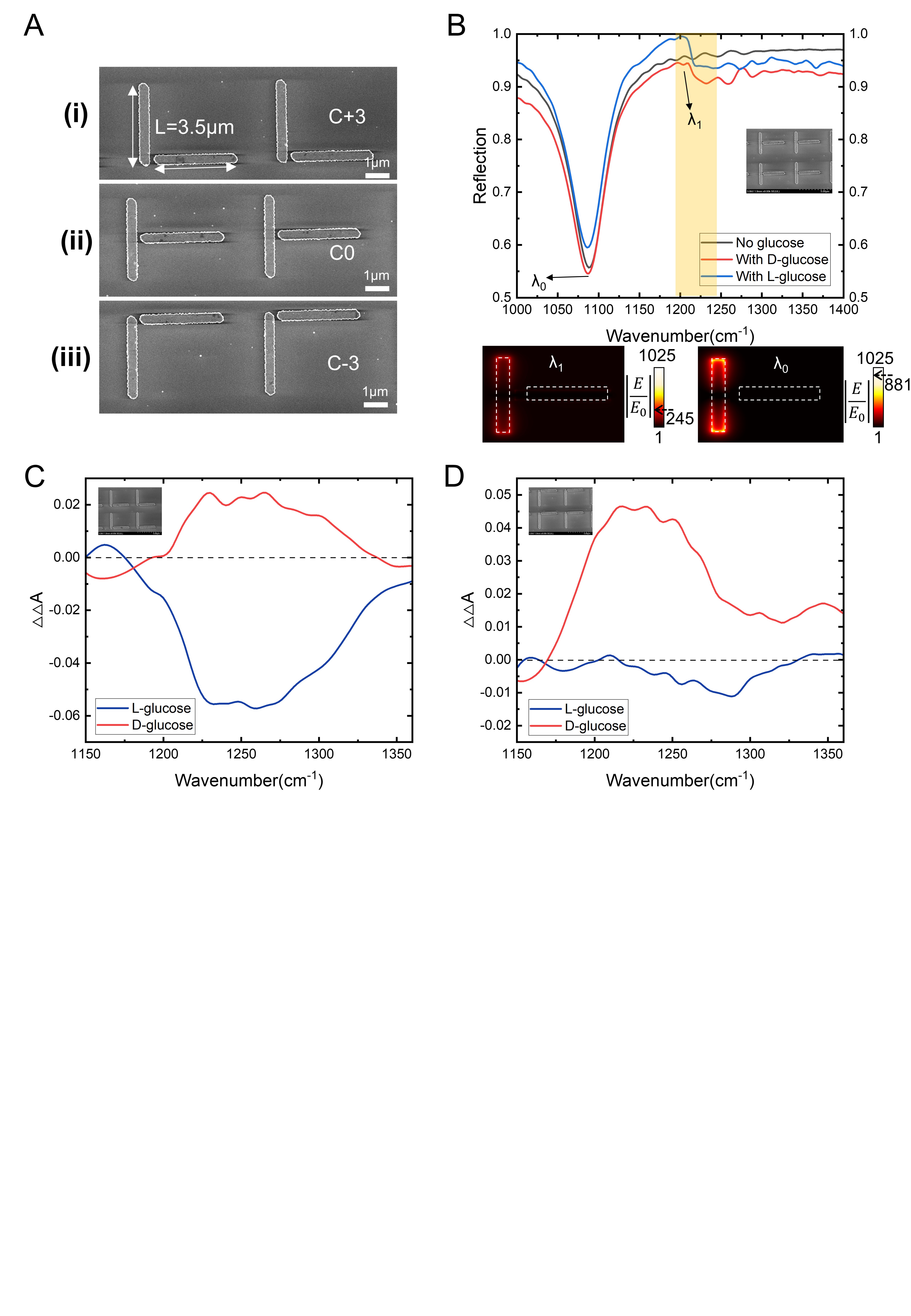
**

**Fig. S17: Demonstration of VCD sensing of D-glucose and L-glucose using the IRCPM platform.** (**A**) (i) to (iii) Fabricated C+3, C0 and C-3 metamaterials with resonance wavelength near 1100 cm^-1^. The length for each nanorod is 3.5 μm. (**B**) Measured IR reflection spectrum of D-glucose and L-glucose coated with C0 metamaterials. Simulation results shows the electric field intensity at two wavelengths. (**C**) Measured enhanced VCD signal of D-glucose and L-glucose coated on C+3 metamaterials. (**D**) Measured enhanced VCD signal of D-glucose and L-glucose coated on C-3 metamaterials.

**
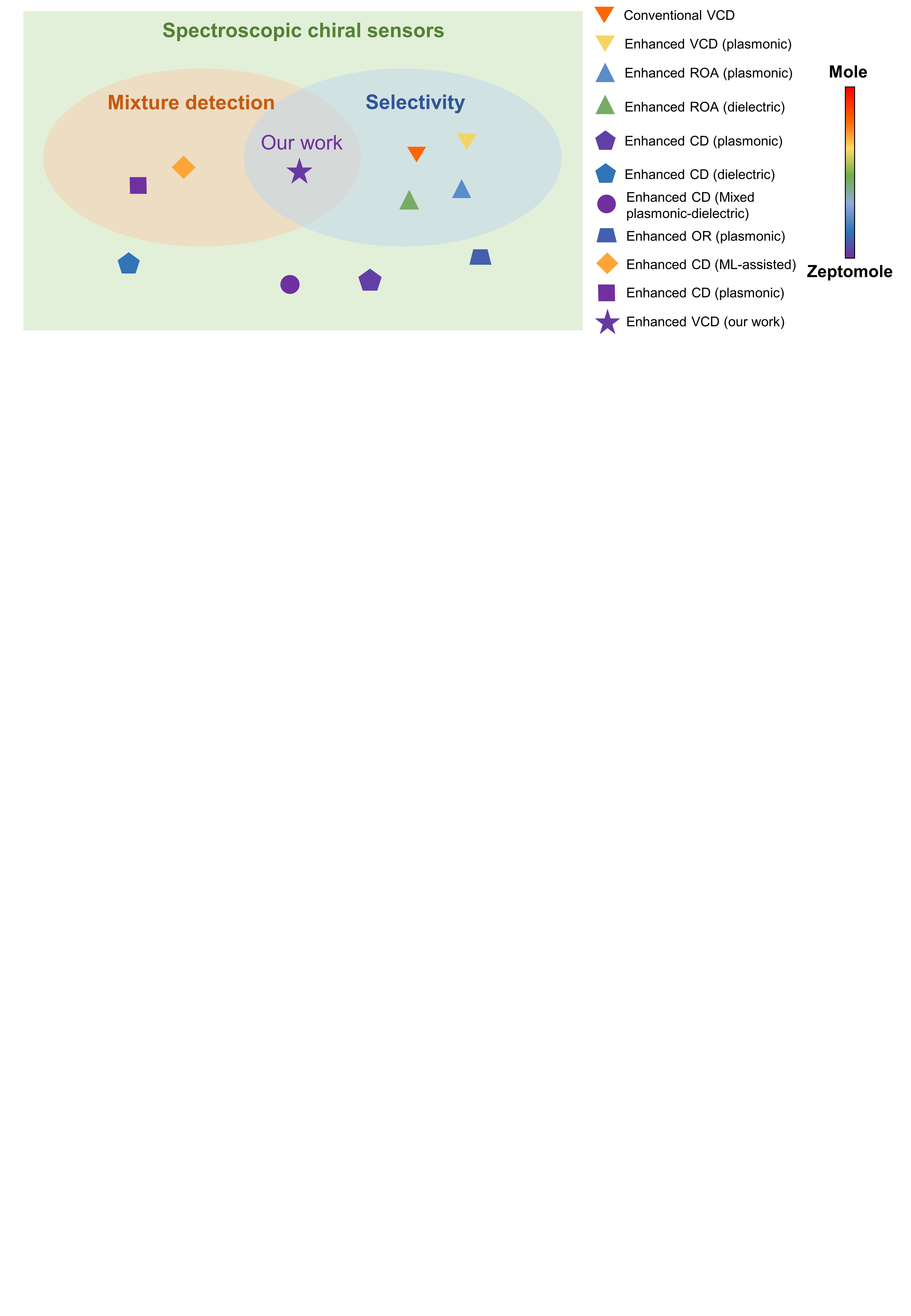
**

**Fig. S18: State-of-the-art spectroscopic chiral sensors. Data are taken from the reference in main text (from top to bottom: Ref. 2, Ref. 40, Ref. S13, Ref. 48, Ref. S7, Ref. S10, Ref. S14, Ref. 39, Ref. 44, Ref. 45)**

**Table S1: Benchmark for chiral sensors**

| Sensing unit | Working wavelength | Enhancement methods | Molecules | Sensitivity | Detection limit | Selectivity | Mixture detection |
| --- | --- | --- | --- | --- | --- | --- | --- |
| VCD spectroscopy^5^ | 6.06 μm & 6.13 μm | N.A. | BSA &β-lactoglobulin | 2.7e-5% per mM & 2.8e-4% per mM | N.A. | Yes | No |
| Gammadion^6^ | 800 nm | Superchiral field | BSA &β-lactoglobulin | 3nm per 15μM & 0.5nm per 54μM | N.A. | No | No |
| Twisted metamaterial^7^ | 1000 nm | Field enhancement | S-1,2-Propanediol & R-1,2-Propanediol | 4deg per 13M & 1deg per 13M | ~55 zeptomole | No | No |
| Crossing slit^8^ | 3.39 μm | Field enhancement | R-α-pinene & S-α-pinene | 2% per 840mg & 3% per 840mg | N.A. | Yes | No |
| Double rod^9^ | 3.23 μm-3.45 μm | Superchiral field | D- analine & L-analine | N.A. | N.A. | Yes | No |
| Achiral metamaterial^10^ | 5.71 μm -6.67 μm | Field enhancement | (d) camphor & (s) camphor | N.A. | N.A. | Yes | No |
| Dielectric resonator^11^ | 700 nm | Field enhancement | L-phenylalanine & D-phenylalanine | 225mdeg per 200nm & 250mdeg per 200nm | N.A. | No | No |
| Microbubble^12^ | 650 nm | Field enhancement | L-glucose and D-glucose | 5nm per 100nM & 5nm per 100nM | <100pM | No | Yes |
| Mechano metamaterial^13^ | 1000 nm | Colloidal assembly | BSA | 28nm per fmol | N.A. | No | No |
| Sub-10nm metamaterial^14^ | 580 nm | Field enhancement | L-cysteine & D- cysteine | 3.7k per mM & 3.5k per mM | N.A. | Yes | No |
| Negative-index metamaterials^15^ | 550 nm | Field enhancement | Flavin mononucleotide | 300 counts per 100nM | ~zeptomole range | No | No |
| Triangular nanorings^16^ | 600 nm -800 nm | Field enhancement | Aβ mono & Aβ fibril | 6.9 a.u./pM | N.A. | Yes | No |
| **Our work** | **5.74 μm-6.67 μm** | **Loss-engineering & Superchiral field** | **BSA &β-lactoglobulin** | **7.61% per μM & 4.00% per μM** | **~ 23 zeptomole** | **Yes** | **Yes** |

**Table S2: Q-factor of chiral metamaterials**

| **Chiral metamaterials** | **C+3** | **C+2** | **C+1** | **C0** | **C-1** | **C-2** | **C-3** |
| --- | --- | --- | --- | --- | --- | --- | --- |
| **Q-factor** | 7.93 | 9.16 | 11.3 | 11.9 | 11.1 | 10.1 | 10.2 |

Supplementary references:

1 Xu J, Ren Z, Dong B, Liu X, Wang C, Tian Y *et al.* Nanometer-Scale Heterogeneous Interfacial Sapphire Wafer Bonding for Enabling Plasmonic-Enhanced Nanofluidic Mid-Infrared Spectroscopy. *ACS Nano* 2020; **14**: 12159–12172.

2 Wei J, Li Y, Chang Y, Hasan DMN, Dong B, Ma Y *et al.* Ultrasensitive Transmissive Infrared Spectroscopy via Loss Engineering of Metallic Nanoantennas for Compact Devices. *ACS Appl Mater Interfaces* 2019; **11**: 47270–47278.

3 Schönhals A, Kröger-Lui N, Pucci A, Petrich W. On the role of interference in laser-based mid-infrared widefield microspectroscopy. *J Biophotonics* 2018; **11**: e201800015.

4 Blasius J, Elfgen R, Hollóczki O, Kirchner B. Glucose in dry and moist ionic liquid: vibrational circular dichroism, IR, and possible mechanisms. *Phys Chem Chem Phys* 2020; **22**: 10726–10737.

5 Baumruk V, Keiderling TA. Vibrational circular dichroism of proteins in water solution. *J Am Chem Soc* 1993; **115**: 6939–6942.

6 Hendry E, Carpy T, Johnston J, Popland M, Mikhaylovskiy R V, Lapthorn AJ *et al.* Ultrasensitive detection and characterization of biomolecules using superchiral fields. *Nat Nanotechnol* 2010; **5**: 783–787.

7 Zhao Y, Askarpour AN, Sun L, Shi J, Li X, Alù A. Chirality detection of enantiomers using twisted optical metamaterials. *Nat Commun* 2017; **8**: 14180.

8 Knipper R, Kopecký V, Huebner U, Popp J, Mayerhöfer TG. Slit-Enhanced Chiral- and Broadband Infrared Ultra-Sensing. *ACS Photonics* 2018; **5**: 3238–3245.

9 Iida T, Ishikawa A, Tanaka T, Muranaka A, Uchiyama M, Hayashi Y *et al.* Super-chiral vibrational spectroscopy with metasurfaces for high-sensitive identification of alanine enantiomers. *Appl Phys Lett* 2020; **117**: 101103.

10 Vázquez-Guardado A, Chanda D. Superchiral Light Generation on Degenerate Achiral Surfaces. *Phys Rev Lett* 2018; **120**: 137601.

11 García-Guirado J, Svedendahl M, Puigdollers J, Quidant R. Enhanced Chiral Sensing with Dielectric Nanoresonators. *Nano Lett* 2020; **20**: 585–591.

12 Liu Y, Wu Z, Kollipara PS, Montellano R, Sharma K, Zheng Y. Label-Free Ultrasensitive Detection of Abnormal Chiral Metabolites in Diabetes. *ACS Nano* 2021; **15**: 6448–6456.

13 Probst PT, Mayer M, Gupta V, Steiner AM, Zhou Z, Auernhammer GK *et al.* Mechano-tunable chiral metasurfaces via colloidal assembly. *Nat Mater* 2021; **20**: 1024–1028.

14 Zhang W, Ai B, Gu P, Guan Y, Wang Z, Xiao Z *et al.* Plasmonic Chiral Metamaterials with Sub-10 nm Nanogaps. *ACS Nano* 2021; **15**: 17657–17667.

15 Indukuri SRKC, Frydendahl C, Sharma N, Mazurski N, Paltiel Y, Levy U. Enhanced Chiral Sensing at the Few-Molecule Level Using Negative Index Metamaterial Plasmonic Nanocuvettes. *ACS Nano* 2022; **16**: 17289–17297.

16 Wang G, Hao C, Ma W, Qu A, Chen C, Xu J *et al.* Chiral Plasmonic Triangular Nanorings with SERS Activity for Ultrasensitive Detection of Amyloid Proteins in Alzheimer’s Disease. *Adv Mater* 2021; **33**: 2102337.
